# Genomic and phenotypic characterization of *Mycobacterium tuberculosis’* closest-related non-tuberculous mycobacteria

**DOI:** 10.1128/spectrum.04126-23

**Published:** 2024-05-03

**Authors:** Camille Sous, Wafa Frigui, Alexandre Pawlik, Fadel Sayes, Laurence Ma, Thomas Cokelaer, Roland Brosch

**Affiliations:** 1 Institut Pasteur, Université Paris Cité, Unit for Integrated Mycobacterial Pathogenomics, CNRS UMR 6047, Paris, France; 2 Institut Pasteur, Université Paris Cité, Plate-forme Technologique Biomics, Paris, France; 3 Institut Pasteur, Université Paris Cité, Bioinformatics and Biostatistics Hub, Paris, France; CNRS-University of Toulouse, Toulouse, France

**Keywords:** *Mycobacterium tuberculosis*, mycobacterial model, virulence, phylotype, evolution, ESX-1

## Abstract

Four species of non-tuberculous mycobacteria (NTM) rated as biosafety level 1 or 2 (BSL-1/BSL-2) organisms and showing higher genomic similarity with *Mycobacterium tuberculosis* (*Mtb*) than previous comparator species *Mycobacterium kansasii* and *Mycobacterium marinum* were subjected to genomic and phenotypic characterization. These species named *Mycobacterium decipiens*, *Mycobacterium lacus*, *Mycobacterium riyadhense,* and *Mycobacterium shinjukuense* might represent “missing links” between low-virulent mycobacterial opportunists and the highly virulent obligate pathogen *Mtb*. We confirmed that *M. decipiens* is the closest NTM species to *Mtb* currently known and found that it has an optimal growth temperature of 32°C–35°C and not 37°C. *M. decipiens* showed resistance to rifampicin, isoniazid, and ethambutol, whereas *M. lacus* and *M. riyadhense* showed resistance to isoniazid and ethambutol. *M. shinjukuense* was sensitive to all three first-line TB drugs, and all four species were sensitive to bedaquiline, a third-generation anti-TB drug. Our results suggest these four NTM may be useful models for the identification and study of new anti-TB molecules, facilitated by their culture under non-BSL-3 conditions as compared to *Mtb. M. riyadhense* was the most virulent of the four species in cellular and mouse infection models. *M. decipiens* also multiplied in THP-1 cells at 35°C but was growth impaired at 37°C. Genomic comparisons showed that the *espACD* locus, essential for the secretion of ESX-1 proteins in *Mtb*, was present only in *M. decipiens*, which was able to secrete ESAT-6 and CFP-10, whereas secretion of these antigens varied in the other species, making the four species interesting examples for studying ESX-1 secretion mechanisms.

**IMPORTANCE:**

In this work, we investigated recently identified opportunistic mycobacterial pathogens that are genomically more closely related to *Mycobacterium tuberculosis* (*Mtb*) than previously used comparator species *Mycobacterium kansasii* and *Mycobacterium marinum*. We confirmed that *Mycobacterium decipiens* is the currently closest known species to the tubercle bacilli, represented by *Mycobacterium canettii* and *Mtb* strains. Surprisingly, the reference strain of *Mycobacterium riyadhense* (DSM 45176), which was purchased as a biosafety level 1 (BSL-1)-rated organism, was the most virulent of the four species in the tested cellular and mouse infection models, suggesting that a BSL-2 rating might be more appropriate for this strain than the current BSL-1 rating. Our work establishes the four NTM species as interesting study models to obtain new insights into the evolutionary mechanisms and phenotypic particularities of mycobacterial pathogens that likely have also impacted the evolution of the key pathogen *Mtb*.

## INTRODUCTION

*Mycobacterium tuberculosis (Mtb),* the etiological agent of human tuberculosis (TB), has evolved as a major human pathogen and continues to be responsible for more than 10 million new TB cases per year and 1.3 million annual deaths (1). The molecular factors and genetic adaptations that have contributed to the emergence of such an outstanding obligate pathogen have only partially been explored. Genomic and phenotypic comparisons with closely related non-tuberculous mycobacteria (NTM) have the potential to uncover specific traits of *Mtb* that might have favored its evolution toward increased pathogenicity. In this respect, closely related opportunistic mycobacterial pathogens, such as *Mycobacterium marinum* and *Mycobacterium kansasii,* were successfully used in the past as models for comparisons (2, 3), but these analyses also revealed a wide evolutionary gap between these two species and *Mtb*, as demonstrated by large genome size differences (6.4/6.6 Mb vs 4.4 Mb) and other variations (e.g., pigmentation in *M. marinum* and *M. kansasii* vs no pigmentation in *Mtb*). However, more recent genome analysis projects have identified several novel mycobacterial species that were closer related to *Mtb* than the previously used comparator models (4). These four species named *Mycobacterium decipiens*, *Mycobacterium lacus*, *Mycobacterium riyadhense*, and *Mycobacterium shinjukuense* were shown to constitute a common clade together with *Mtb* that was named *Mtb*-associated phylotype (MTBAP) (5). Further genomic analyses showed that MTBAP members shared with *Mtb* many attributes that might be linked with adaptation to special environments, such as those found inside host cells, suggesting that certain host adaptation processes emerged in members of this phylotype already before the speciation of *Mtb* (5).

These four MTBAP members thus represent interesting new mycobacterial study models that share many important features with *Mtb* but, at the same time, have advantageous characteristics compared to *Mtb* strains. While *Mtb* is a BSL-3 pathogen, the other four MTBAP members were described as BSL-1 or BSL-2 organisms, a finding that facilitates their handling in the laboratory. As it is important for research to have efficient and predictive study models available, we investigated selected features of the four MTBAP members and, more generally, the individual suitability of one or more of these species to serve as an accurate mycobacterial study model.

## RESULTS

### *In vitro* growth assays

**Fig 1:**
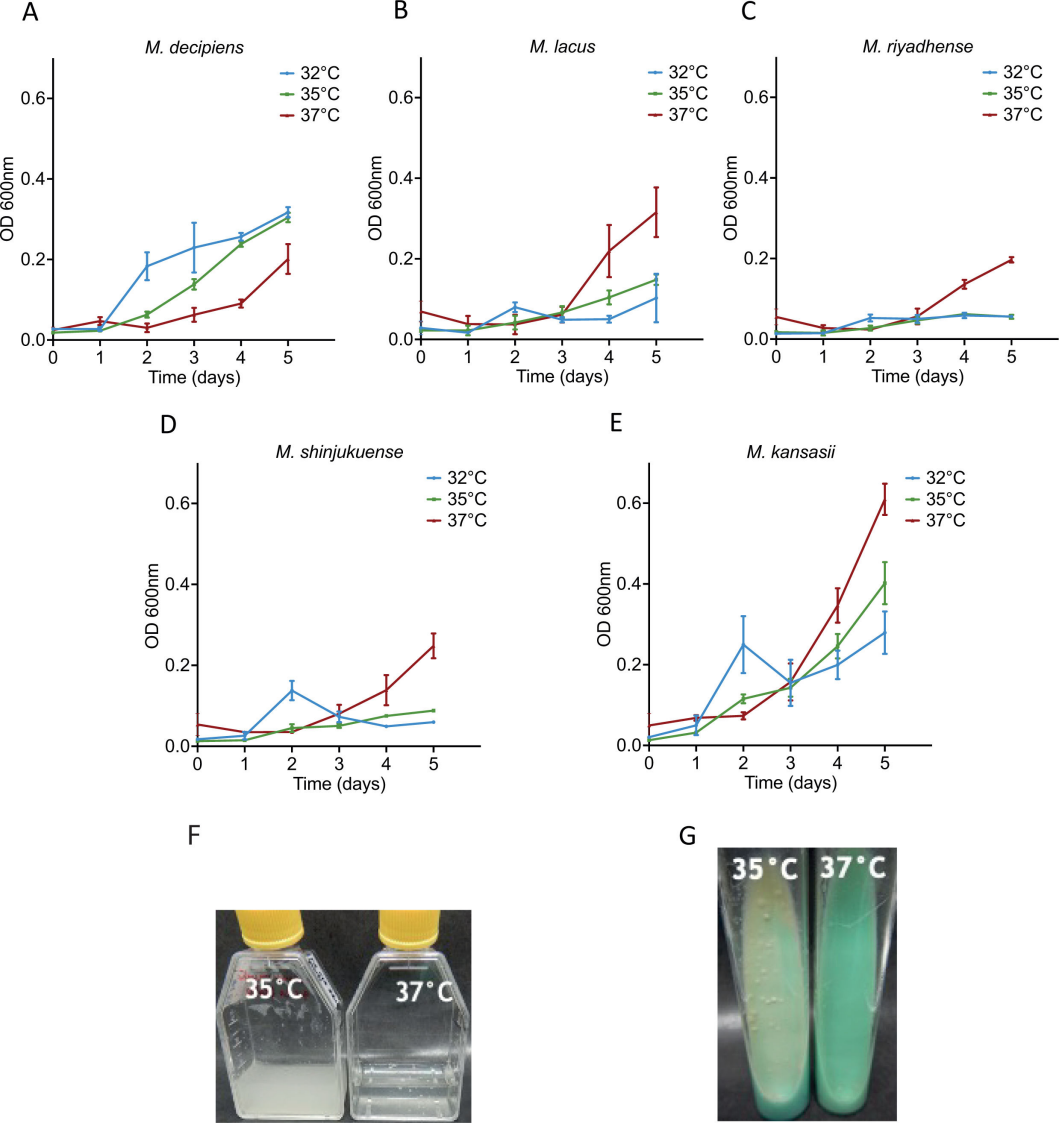
Growth measurement over time at an optical density of 600 nm for *M. decipiens* (**A**), *M. lacus* (**B**), *M. riyadhense* (**C**), *M. shinjukuense* (**D**), and *M. kansasii* (**E**). Growth was measured at 32°C, 35°C, and 37°C for each strain. Results from two independent experiments in classic culture conditions. Picture of *M. decipiens* growth at 35°C and 37°C in 7H9 ADC liquid cultures (**F**) and on LJ tubes (**G**) were taken after 10 days of incubation. Comparable quantities of bacteria were used for both temperatures.

Four reference strains from the recently described mycobacterial species *M. decipiens*, *M. lacus*, *M. riyadhense*, and *M. shinjukuense* were purchased from culture collections and cultivated according to the suppliers’ instructions, which indicated an optimal growth temperature of 37°C in mycobacterial standard media for each of the strains. During our initial attempts to culture the strains, we observed a growth limitation for *M. decipiens* at 37°C, which resulted in an extremely low yield in liquid or solid media even in cultures that were left to grow for over 2 months. This observation prompted us to determine the growth rates of the four species and one *M. kansasii* control strain at 32°C, 35°C, and 37°C. Measurements of the optical density (OD) at 600 nm were performed every 24 hours for 5 days before formation of clumps. As shown in Fig. 1, *M. lacus*, *M. riyadhense*, *M. shinjukuense*, and *M. kansasii* showed better growth at 37°C than at 35°C and 32°C, whereas *M. decipiens* grew faster at 32°C and 35°C than at 37°C. We concluded that *M. decipiens* had an optimal growth temperature of 35°C and that it behaved differently in our settings than indicated by the information supplied by the culture collection from where the strain was obtained. This information was important for all further experiments as *M. decipiens* cultures were then routinely tested at 35°C in addition to 37°C.

### Genomics

The assemblies of the long-read data were performed with the long-read assembly pipeline (LORA) from the Sequana project (6). Starting from the raw data, it utilizes the Canu (7) assembler and a set of quality metrics to check the completeness of the final assemblies. Assembly of the *M. riyadhense* long-read data generated by PacBio Sequencing showed the presence of three contigs, which is consistent with existing knowledge for this bacterium, as two of the contigs correspond to plasmids (8). In our assembly, the size of the whole chromosome measured 6,155,284 bp, whereas the size of the two plasmids ranged between 472,572 bp (pMR1_45176) and 7,354 bp (pMR2_45176). Sequencing of *M. shinjukuense* revealed a chromosome size of 4,489,120 bp and no plasmid, whereas initial assembly of the *M. decipiens* data generated two contigs, one large one (5.3 Mb) and one small one (17 kb). Closer analysis using PCR amplification approaches with selected primers showed that the smaller contig, which largely overlapped with the large contig, did not correspond to a plasmid or extrachromosomal element but was part of the chromosome. The apparent reason why the assembly program generated an extra contig was due to a small region of difference of ~2 kb inside this part of the genome that was deleted in the genomes of a subfraction of the bacteria used to prepare the genomic DNA from. Further PCR tests on fresh cultures of *M. decipiens* revealed that the deletion was no longer found in these younger cultures. We conclude from these results that *M. decipiens* is characterized by a single chromosome with a length of 5,303,299 bp and contains no plasmid (Table 1).

**TABLE 1:** Genome sizes of the four NTM and control strains

Name	Designation	Chromosome size	Plasmid designation	Plasmid sizes
*M. decipiens*	ATCC TSD-117	5,303,299 bp	–^*a*^	–
*M. lacus*	DSM 44577	5,077,830 bp	–	–
*M. riyadhense*	DSM 45176	6,155,284 bp	pMR1_45176 pMR2_45176	472,572 bp 7,354 bp
*M. shinjukuense*	DSM 45663	4,489,120 bp	–	–
*M. kansasii*	ATCC 12478	6,432,277 bp	pNC_022654	144,951 bp
*M. marinum*	M	6,636,827 bp	pNC_010604	23,317 bp
*M. tuberculosis*	H37Rv	4,411,532 bp	–	–

^*a*^ "-" : not applicable.

To confirm these conclusions, additional new DNA preparations were subjected to Nanopore long-read sequencing. Data obtained from this approach confirmed our initial data of a single circular chromosome for *M. decipiens*. Deposit of the long-read sequence data in the European Nucleotide Archive has then been effectuated.

### Comparison of selected genomic regions

**Fig 2:**
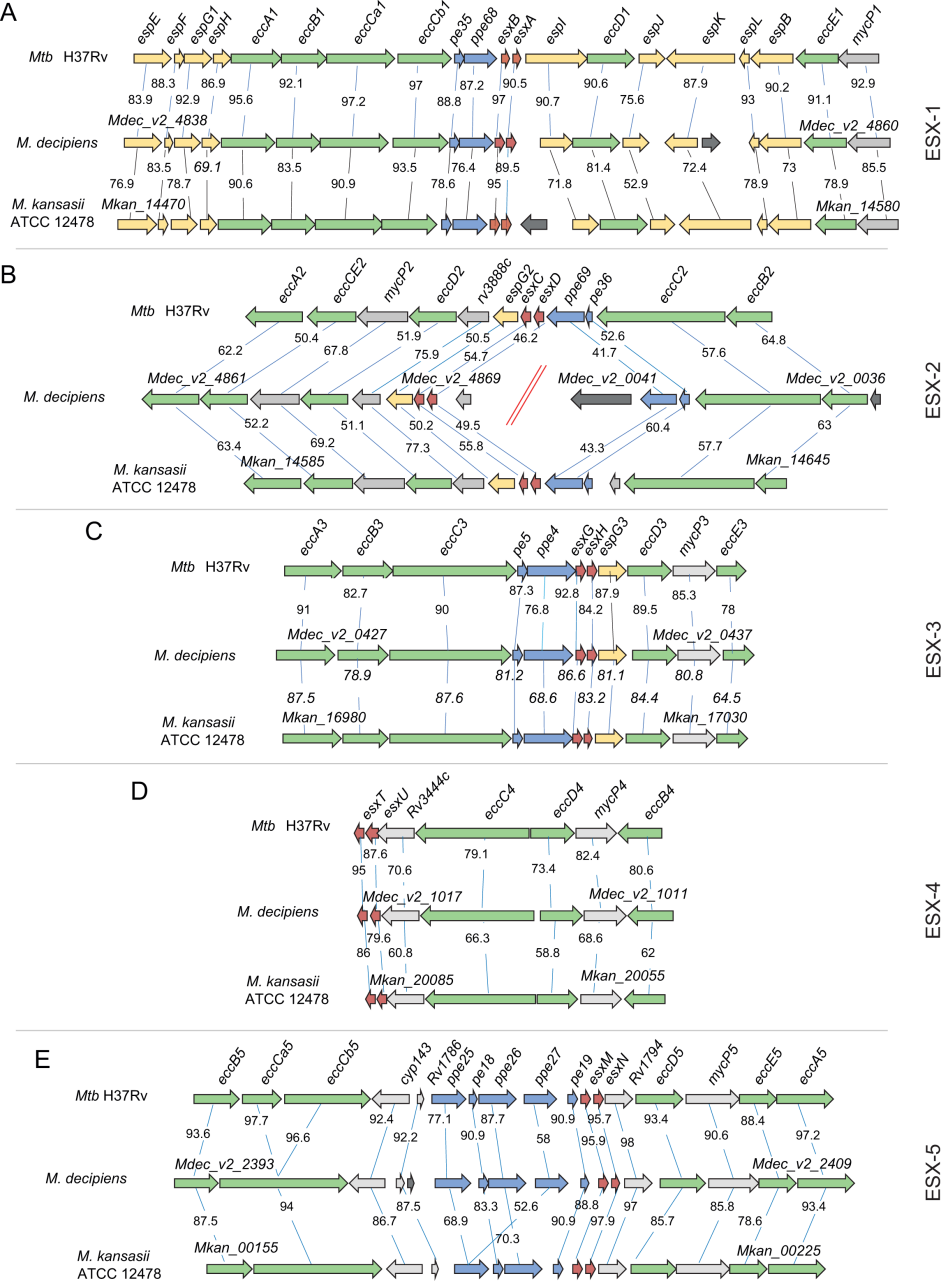
Genomic comparisons of ESX loci of *M. decipiens* with *Mtb* and *M. kansasii*. Comparisons of ESX-1 (**A**), ESX-2 (**B**), ESX-3 (**C**), ESX-4 (**D**), and ESX-5 (**E**) were performed using the Artemis Comparison Tool ACT, and the “MicroScope” database from the Genoscope (https://mage.genoscope.cns.fr/microscope/mage/viewer.php?).

To allow further comparisons, the assembled sequences were then uploaded into the Microbial Genome Annotation and Analysis platform “MicroScope” (9), which allowed comparisons of genomic regions of interest to be undertaken. For *M. lacus*, for which our initial PacBio-generated long-read-based sequencing approach was not successful due to low DNA quality of the preparation, an independent second DNA preparation and long-read sequencing approach generated a single contig of 5,077,830 bp. For the comparative analyses of this species, as well as for the reference comparator species *M. kansasii* and *Mtb*, we initially used the genome sequences available in the NCBI database. We were particularly interested in the genomic regions that encode ESX type VII secretion systems, several of which are known to impact mycobacterial virulence (10), in order to determine whether these chromosomal regions of the four MTBAP members were closer related to *Mtb* than to *M. kansasii* or vice versa. Figure 2 shows an example of the results obtained for the genomic organization of the five ESX loci of *M. decipiens* that were compared with those of *Mtb* and *M. kansasii*. In Table 2, the average locus similarity expressed as the amino acid identity is shown. Moreover, comparisons were also made for the ESX loci of the three other species, as depicted in Fig. S1A through E. These analyses revealed that apart from the ESX-2 locus, the predicted proteins of the four other ESX loci of *M. decipiens* (ESX-1, ESX-3, ESX-4, ESX-5) share the highest amino acid identity percentages with their orthologs from *Mtb*. For example, *M. decipiens* ESX-1 core proteins that build the ESX-1 secretion apparatus, such as the EccB, EccCa_1_, EccCb_1_, EccD_1_, EccE_1_, and MycP_1_ proteins, show between 90.6 and 97.2 percent sequence identity with the orthologous proteins of *Mtb*, which is higher than that of the other species, including *M. kansasii* (Fig. 2A). These findings correlate well with the overall very close genomic relatedness of *M. decipiens* and *Mtb*, which can also be observed in most other ESX systems, such as ESX-3, ESX-4, and ESX-5. While the genomic organization in the ESX-3 and ESX-4 loci was highly conserved among *Mtb*, *M. decipiens,* and *M. kansasii*, some differences were noticed for ESX-5. The first difference concerns the orthologs of *Mtb* H37Rv genes *eccCa_5_* and *eccCb_5_*, which are represented as single genes in *M. decipiens* and *M. kansasii* (Fig. 2). However, this discrepancy is due to a sequencing error in the original *Mtb* H37Rv genome sequence (A instead of T at position 2020563), as previously reported (11). Other potential differences concern the number of *ppe* genes in the *pe* and *ppe* gene locus of ESX-5. In *Mtb* H37Rv and *M. decipiens,* this site harbors three *ppe* genes (*ppe25*, *ppe26,* and *ppe27*) and two *pe* genes (*pe18* and *pe19*), whereas in *M. kansasii,* only two *ppe* (*ppe25* and *ppe26*) and two *pe* (*pe18* and *pe19*) orthologs are present, which also corresponds to the situation observed in *M. lacus* and *M. shinjukuense* (Fig. S1E). Interestingly, *M. riyadhense* shows two pairs of *ppe25* and *ppe26* homologs and two *pe* (*pe18* and *pe19*) genes in this locus, whereby the first *ppe* pair shows higher similarity with *Mtb ppe25* and *ppe26* genes than the second pair (Fig. S1E). It seems that this part of the ESX-5 locus is subject of substantial variations, which likely were caused during mycobacterial evolution by gene duplications of *pe* and *ppe* genes that are present in other ESX-loci only as single *pe-ppe* pairs (ESX-1, ESX-2, and ESX-3). This process might have been combined with putative events of horizontal gene transfer, as seen for *ppe27*, for which orthologs are present only in *Mtb* and *M. decipiens* and not in the other studied species.

**TABLE 2:** Average of amino acid identity of the ESX systems for the four strains compared to *Mtb* and *M. kansasii^a^*^*b*^

	ESX-1		ESX-2		ESX-3		ESX-4		ESX-5	
	*Mtb* H37Rv	*M. kansasii*	*Mtb* H37Rv	*M. kansasii*	*Mtb* H37Rv	*M. kansasii*	*Mtb* H37Rv	*M. kansasii*	*Mtb* H37Rv	*M. kansasii*
*M. kansasii*	79.8	–	87.7	–	80.9	–	68.8	–	86.5	–
*M. decipiens*	90.2	79.5	56.4	57.8	86.0	80.7	81.3	68.9	90.4	86.9
*M. lacus*	81.1	84.4	90.2	89.1	81.7	81.9	71.2	67.7	86.8	87.2
*M. riyadhense*	78.8	83.7	56.2	57.3	82.8	82.4	71.6	69.1	85.9	84.6
*M. shinjukuense*	80.5	84	90.2	86.7	81.3	82.0	72.4	68.9	87.0	87.8

^*a*^ Percentages were determined using Mage software from the Genoscope.

^*b*^ "-" : not applicable.

As mentioned above, ESX-2 is an exception in terms of ortholog protein similarity, as the predicted proteins encoded in the ESX-2 locus of *M. decipiens* showed 50.4%–67.8% sequence similarities with ESX-2 Ecc and MycP proteins of *Mtb* and thus much lower values than seen for other ESX loci (Fig. 2B). In addition, the genes of the ESX-2 system were assigned to two distinct sections of the *M. decipiens* genome (Fig. 2B), a split that was confirmed with PCR amplification experiments using specific primers (Fig. S2). Interestingly, the ESX-2 locus of *M. riyadhense* showed a similar separation into two distinct genomic areas and low protein similarities with ESX-2 proteins (Fig. S1B). While ESX-2 systems belong to the evolutionary youngest ESX systems that are found only in selected slow-growing mycobacterial species (12, 13), the biological role of the ESX-2 secretion system in mycobacteria remains poorly explored. The observed heterogeneity of the ESX-2 systems adds new aspects to the research on the putative origin and function of ESX-2 systems, with *M. decipiens* and *M. riyadhense* as new study models for this evolutionary pertinent question.

### Assessment of ESX-1 type VII secretion activity

The ESX-1 type VII protein secretion system is an important virulence determinant for *Mtb* and several other pathogenic slow-growing mycobacteria. The best characterized ESX-1 secretion substrates are the 6-kDa early secretory antigenic target ESAT-6 (also known as EsxA) and the 10-kDa culture filtrate protein CFP-10 (also known as EsxB), which have been extensively studied and are also known to be implicated in phagosomal rupture during *Mtb* infection (10, 14, 15). We thus included the assessment of the production and secretion of ESAT-6 and CFP-10 into the phenotypic characterization scheme of the four mycobacterial species and performed western blot analyses using polyclonal anti-ESAT-6 and anti-CFP-10 antibodies for the detection of the two proteins in whole-cell lysate (WCL) and culture supernatant fractions. In addition, anti-SigA was used as lysis control. We also included anti-Ag85B control antibodies to evaluate the potential presence of secreted proteins by the twin-arginine translocation system.

**Fig 3:**
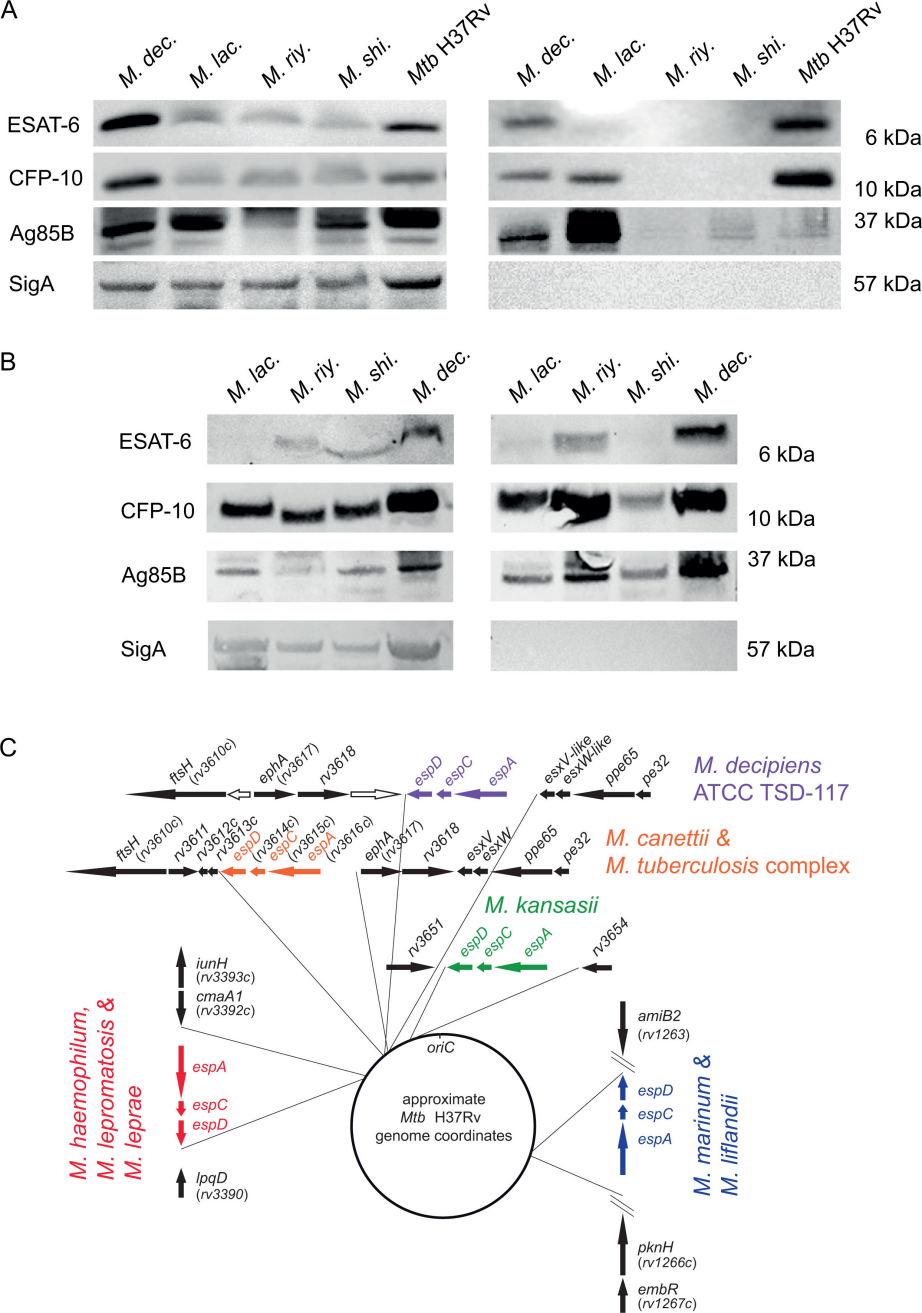
Western blot-based evaluation of the presence of ESAT-6 and CFP-10 in whole-cell lysate (left panel) and supernatant (right panel) fractions of *in vitro* cultures of *M. decipiens* (*M. dec*.), *M. lacus* (*M. lac*.), *M. riyadhense* (*M. riy*.), *M. shinjukuense* (*M. shi*.), and *Mtb* H37Rv that were grown to an OD_600nm_ of 0.6–0.8 using anti-ESAT-6, anti-CFP-10, as well as anti-Ag85B and anti-SigA control antibodies. An amount of 50 µg of proteins was migrated on SDS-PAGE for 30 min at 200 V and transferred onto a nitrocellulose membrane for 7 min at 110 V (**A**). Western blotting of whole-cell lysate (left panel) and supernatant (right panel) fractions from *M. lacus* (*M. lac*.), *M. riyadhense* (*M. riy*.), *M. shinjukuense* (*M. shi*.), and *M. decipiens* (*M. dec*.) cultures that were grown to an optical density higher than 1 (**B**). Genomic position of the orthologous genes of the *espACD* cluster that is present in the genome of *M. decipiens*, compared to the genomic locations of orthologous *espACD* clusters in selected other mycobacterial species that possess an *espACD* cluster in their genomes (**C**). Note that the orthologous flanking genes of the *espACD* cluster identified in the various species refer to the *Mtb* H37Rv gene nomenclature and relative genomic localization. Image adapted from reference (16) with permission and complemented with new information on the *M. decipiens espACD* cluster. Genes depicted as white arrows correspond to *M. decipiens* specific genes.

In initial experiments, we grew the strains to an OD_600nm_ of 0.6–0.8, commonly used for immuno-blot analyses involving *Mtb*. Equal amounts of total protein were loaded for the WCL and culture supernatant fractions of each strain. Inspection of the WCL fractions showed the production of both ESAT-6 and CFP-10 in all tested strains (Fig. 3A). The overall identification of the two proteins in the WCL fraction is consistent with the presence of the *esxA* and *esxB* orthologs in the genomes of the four strains (Fig. 2A; Fig. S1A). For the supernatant fractions, we noticed that CFP-10 secretion was detected for *M. decipiens*, *M. lacus,* and *Mtb*, whereas ESAT-6 secretion was only detected for *M. decipiens* and the *Mtb* H37Rv control strain. It is noteworthy that despite loading comparable amounts of total protein, we did neither detect ESAT-6 nor CFP-10 in the culture supernatants of *M. riyadhense* and *M. shinjukuense,* and there was also no or very little Ag85B detected in the supernatants of these strains (Fig. 3A). To further explore this surprising lack of secretion activity of *M. riyadhense* and *M. shinjukuense*, in additional experiments, we grew the cultures of the four NTM strains to higher ODs >1, which showed that under these conditions, the secretion profiles were different than the ones obtained from cultures harvested at OD 0.6–0.8. Indeed, under these conditions, we observed the presence of CFP-10 in the supernatants of all four NTM, whereas ESAT-6 was detected for *M. decipiens*, and at low amounts also for *M. riyadhense* (Fig. 3B). Importantly, the detection of these antigens was apparently not caused by bacterial lysis as indicated by the SigA lysis control, which remained negative even for supernatants of the cultures grown to higher ODs (Fig. 3B).

Our findings suggest that ESX-1 secretion profiles in *in vitro* cultures of these NTM depend on the species/strain, as well as on the culture densities from which the samples were taken. It is thus likely that besides the genome organization of the ESX-1 core locus, additional factors might interfere with the secretion phenotype. One such factor might be the presence or absence of the *espACD* locus, described as a gene cluster outside the ESX-1 core region, which is encoding ESX-1-associated proteins in *Mtb* that show secretion inter-dependence with EsxA and EsxB (17, 18).

Inspection of the genome sequences of the four strains showed that an *espACD* gene cluster was only present in *M. decipiens* but not in the other three NTM strains of our study. We found that the *espACD* locus in *M. decipiens* was localized in a similar genomic region as its orthologous counterpart in the *Mtb* genome (Fig. 3C) but that it was inserted three genes downstream relative to *Mtb* and *Mycobacterium canettii* genomes, which share an identical *espACD* insertion site. Indeed, as depicted in Fig. 3C, the *espACD* genes of *M. decipiens* are situated between the orthologs of *ephA* (*rv3817*), *rv3618,* and an *M. decipiens*-specific gene coding for an *M. decipiens*-specific oxidoreductase on the one side and neighboring *esxN-*like, *esxP*-like, *ppe65,* and *pe32* orthologs on the other side. We also found that the *espACD* operon was flanked upstream by a genomic region that showed no probable open reading frame, similar to the region upstream of *espACD* in *Mtb*, which contains the DNA-binding regions of several two regulatory systems, including EspR and MptAB (16). These observations suggest that the *espACD* operon of *M. decipiens* was likely introduced into the genome of *M. decipiens* via horizontal gene transfer during the evolution of this species. As the insertion site is close but still different from one of the *Mtb* and *M. canettii* strains, and also differs from the various insertion sites of *espACD* loci in other pathogenic slow-growing mycobacteria (16), it seems likely that the *espACD* locus represents a mycobacterial genomic island that can integrate into the genomes of selected mycobacterial species at different genomic sites and whose function seems to be associated with efficient secretion of ESAT-6 (EsxA), as observed here for *M. decipiens*.

### Assessment of selected antibiotic resistance profiles

Knowledge on the antibiotic resistance profiles constitutes an important information for the phenotypic characterization of bacterial strains and species and the evaluation of their potential usefulness as TB infection models. As such, three first-line anti-TB agents (isoniazid, rifampicin, and ethambutol) and one new generation third-line agent (bedaquiline) were selected to be tested on *M. decipiens*, *M. lacus*, *M. riyadhense*, and *M. shinjukuense*. These experiments based on colorimetric evaluation (resazurin tests) revealed minimal inhibitory concentrations (MIC) that are listed in Table 3 (and in Fig. S3—resazurin plates). We used concentrations that approximated the MIC commonly applied for evaluation of resistance of *Mtb* strains to suggest resistance or susceptibility to the four anti-TB drugs in the tested species. As listed in Table 3, based on comparison with *Mtb*, we found that three of the four species showed intermediate resistance to ethambutol and isoniazid, with *M. shinjukuense* being the sensitive exception. Moreover, *M. decipiens* showed intermediate resistance to rifampicin, whereas the other three species appeared sensitive to that first-line anti-TB drug. Finally, all tested species were susceptible to bedaquiline, a drug that was recently included into TB drug regimens against MDR *Mtb* strains (19, 20).

**TABLE 3:** Determination of MIC range by resazurin assay^*a*^

	*M. decipiens*	*M. lacus*	*M. riyadhense*	*M. shinjukuense*
Bedaquiline	0.0125–0.025 (S)	0.0125–0.025 (S)	0.0125–0.025 (S)	0.00625–0.0125 (S)
Ethambutol	7.5–15 (R)	3.75–7.5 (R)	3.75–7.5 (R)	1.875–3.75 (S)
Isoniazid	1–2 (I)	4–8 (R)	4–8 (R)	0.25–0.5 (S)
Rifampicin	0.5–1 (I)	0.125–0.25 (S)	0.0625–0.125 (S)	0.0156–0.0313 (S)

^*a*^ Concentrations are in µg/mL. Experiments were carried out in four biological replicates with technical duplicates for each experiment. Resistance or susceptibility of the species is indicated in the table and was based on *Mtb* MIC values. I, intermediate; R, resistant; S, susceptible.

The finding that isoniazid and ethambutol showed only little activity on *M. decipiens*, *M. lacus*, and *M. riyadhense* prompted us to investigate this question in more detail. It is true that *Mtb* is particularly sensitive to isoniazid, compared to other mycobacteria, such as *Mycobacterium smegmatis,* as isoniazid is a prodrug that needs to be converted inside the bacterium into the active molecule. In *Mtb*, this activation process is achieved by KatG, an enzyme with catalase and peroxidase activity, whereby the active molecule acts on InhA (21, 22). We therefore analyzed the genomic sequences of the three strains for the presence of *katG* and *inhA* orthologs. The KatG mutation most commonly causing *Mtb* resistance is the mutation of Ser315Thr (23). This mutation is not found in any of the three species, but we observed numerous other mutations in the KatG protein sequence of the three species, some of which might be involved in resistance, although such an effect has not yet been subject of phenotypic evaluation.

Concerning ethambutol, this anti-TB drug blocks the synthesis of arabinogalactan, an important structural element in the mycobacterial cell envelope. There are three mutations in the *embB* gene that are the main cause of resistance to ethambutol in *Mtb* (24, 25). One of these, targeting codon Gly406, also affects *M. lacus*, which shows a Gly406Pro mutation in EmbB, although it is different to the amino acid change found in *Mtb* (Gly406Ala). Overall, the orthologous regions of the 597-bp-sized ethambutol resistance determining region in *Mtb* show several mutations in *M. decipiens*, *M. lacus*, and *M. riyadhense*, but none of which appears to be shared across the three species (26).

Finally, given the putative intermediate resistance of *M. decipiens* to rifampicin, we also analyzed the sequence of *rpoB*, coding for the beta subunit of the RNA polymerase, which represents the target of rifampicin (27). Inspection of RpoB showed that the known *Mtb* mutations causing rifampicin resistance (22) were not found in the RpoB sequence of *M. decipiens*. However, additional genome analysis revealed the presence of an *arr* gene encoding a rifampin ADP-ribosyl transferase in the genome of *M. decipiens* that predicts 78.9% amino acid identity with the orthologous enzyme in *M. smegmatis*. At present, it remains unknown whether the presence of such a gene in the genome of *M. decipiens* might be causing the observed intermediate resistance.

### Assessment of virulence in the THP-1 macrophage infection model

Our phenotypic characterization approach of the four mycobacterial species also included the evaluation of their capacity to multiply in host cells. To best match the optimal growth temperature of *M. decipiens*, infection experiments were carried out both at 35°C and 37°C for 7 days, first removing extracellular bacteria. The capacity for intracellular growth was determined by counting CFUs after 0, 3, 5, and 7 days. As initial dose for infection, a multiplicity of infection (MOI) of 1 CFU per 20 macrophages (1:20) was chosen, based on previous results with *Mtb* that usually allow host cells to survive for about 7 days. In addition, we used the attenuated *Mycobacterium bovis* BCG Pasteur vaccine strain as control, as this strain commonly does not multiply in host cells.

**Fig 4:**
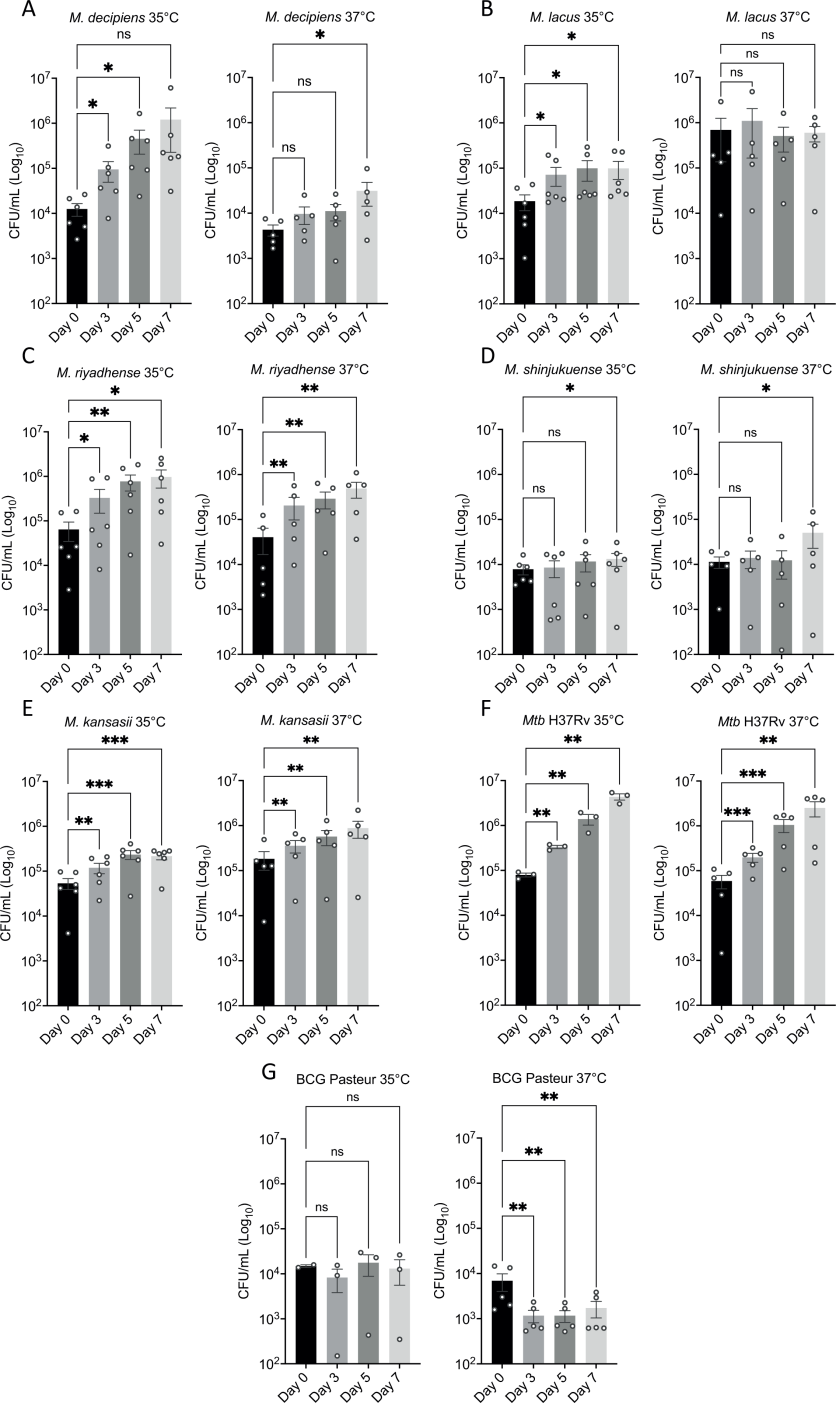
Infection of THP-1-derived macrophages determined by CFU counting at 0, 3, 5, and 7 days after infection. Results represent between three and six independent experiments at 35°C and 37°C for *M. decipiens* (**A**), *M. lacus* (**B**), *M. riyadhense* (**C**), *M. shinjukuense* (**D**), *M. kansasii* (**E**), *M. bovis* BCG Pasteur strain (**F**), and *Mtb* H37Rv (**G**). Data are represented as means and SEM of three to six biological experiments, each experiments containing three technical replicates. Statistical differences in CFU were determined by a two-way ANOVA test with Dunnett’s multiple comparisons test (*P < 0.033; **P < 0.002; ***P < 0.001; ns, non-significant).

Our results confirmed that *Mtb* multiplies in THP-1 cells at 37°C and 35°C, in both cases with a two-log increase of the bacterial load over 7 days (Fig. 4G). The results also showed that *M. kansasii* multiplied in THP-1 cells but at a lower rate than *Mtb*, showing less than one-log increase of CFU counts in 7 days (Fig. 4E), while BCG Pasteur CFU counts slightly decreased, consistent with its virulence attenuation (Fig. 4F).

Intracellular CFU counts for *M. lacus* and *M. shinjukuense* remained stable over the time course of the experiment (Fig. 4B and D). Finally, *M. decipiens* and *M. riyadhense* were able to multiply at both temperatures, with more intracellular growth of *M. decipiens* at 35°C than at 37°C, a finding likely linked to the growth limitation of *M. decipiens* at 37°C, observed in *in vitro* screens (Fig. 1A, F through G and Fig. 4A). Conversely, *M. riyadhense* showed an increase in CFU counts at both temperatures, with an increase of 1–1.5 log in 7 days (Fig. 4C).

### Assessment of virulence in mouse infection models

While cellular infection experiments have the potential to examine intracellular bacterial growth, they only partially reflect the ability of bacteria to multiply in a complex host. To determine growth characteristics inside a mammalian host in the presence of innate and adaptive immune systems, murine models provide valuable additional information. We thus initiated pilot experiments using two different mouse models. We chose C3HeB/FeJ mice which are more susceptible to TB as well as C57BL/6J mice, which are commonly more resistant to TB as models, and subjected groups of mice to aerosolized suspensions of *M. decipiens*, *M. lacus*, *M. riyadhense*, *M. shinjukuense*, *M. kansasii*, *M. bovis* BCG, and *Mtb* H37Rv in a custom-made aerosolization apparatus inside the BSL-3 animal facility. For these initial experiments, solutions with a target concentration at 2.5 × 10^6^ CFUs/mL were prepared, of which 5 mL was loaded into the aerosol-producing devices. From previous experiments with *Mtb,* it was known that such a concentration generates a low-dose infection of approximately 10–50 CFUs per lungs in mice with *Mtb*.

**Fig 5:**
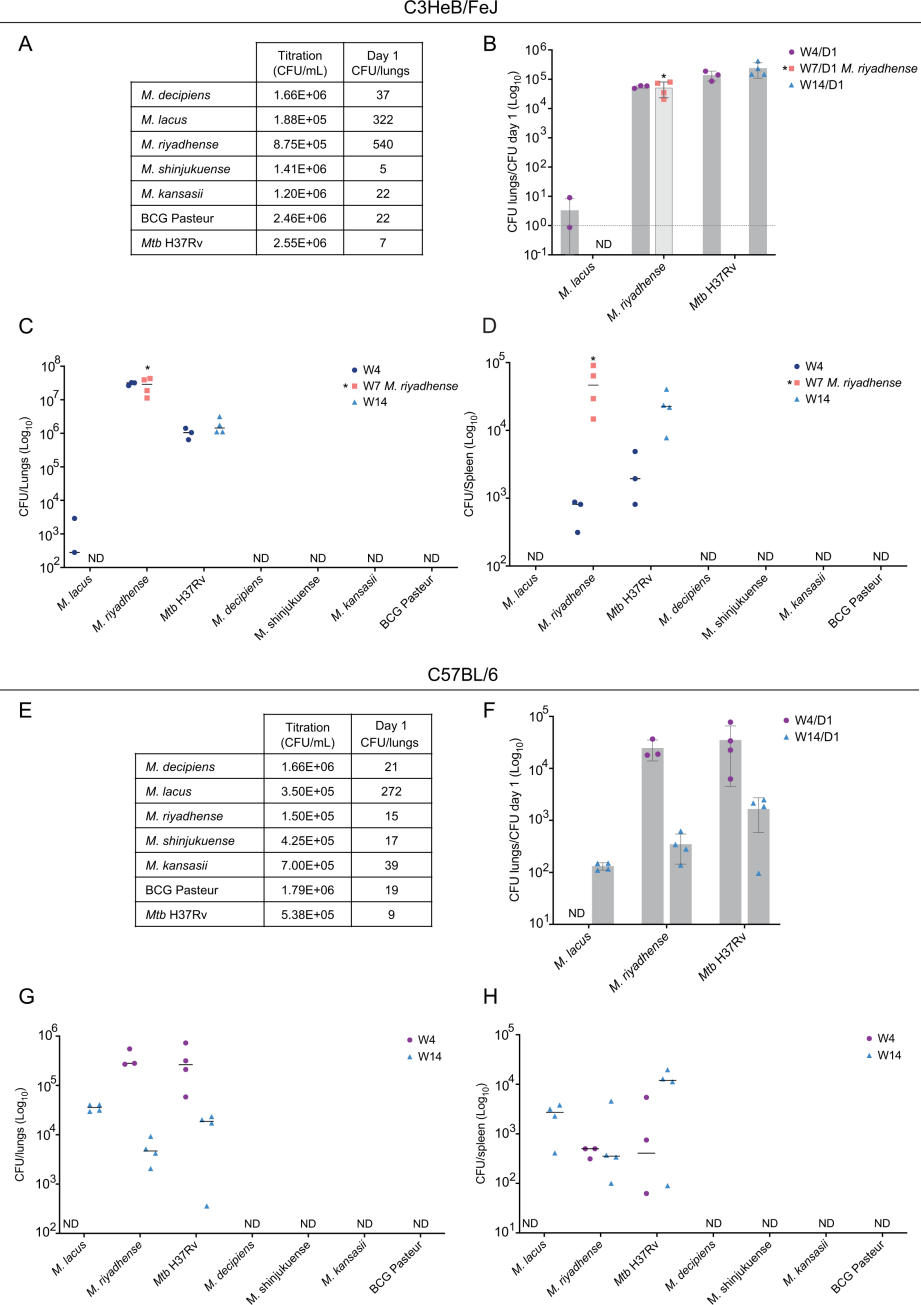
Evaluation of *in vivo* growth capacity of *M. decipiens*, *M. lacus*, *M. riyadhense*, *M. shinjukuense*, *M. kansasii*, *M. bovis* BCG Pasteur, and *Mtb* H37Rv in two murine models. CFU counts in control mice at day 1 post-infection and titration of aerosolized culture of both experiments are indicated (A and E). Results from C3HeB/FeJ infection and ratios of different time points vs day 1 counts are indicated (B). Results from C3HeB/FeJ infection based on CFU counts at different time points. Results are shown at a log_10_ scale for lungs (C) and spleens (D). Same types of results are presented for the C57BL/6J infection with ratios of CFUs D1/CFUs titration (F), CFU counts in lungs (G) and spleens (H). Each experiment was performed once, and the represented values correspond to the number of mice at each time point. "ND": not detected.

As regard the first experiment involving C3HeB/FeJ mice, lung CFU counts from control mice culled at day 1 post-infection showed that for most strains, the low-dose target CFU count was obtained (Fig. 5A), whereas CFU counts for *M. riyadhense* and *M. lacus* were higher. It is likely that these higher numbers have also influenced the relative *in vivo* growth characteristics compared to the other tested species. While the day 1 CFU counts confirmed that mice from all groups contained some of the delivered mycobacteria in their lungs (Fig. 5A and E), we noted that, at weeks 4 and 14 post-infection, the CFU counts of *M. decipiens*, *M. shinjukuense*, *M. kansasii,* and the BCG Pasteur strain were below detection level in organs of C3HeB/FeJ and C57BL/6J mice (Fig. 5C, D, G, and H). We assume that in contrast to *Mtb*, which showed low day 1 CFU counts but then increased CFU counts over time, the low-dose infection with these four strains was controlled by the mice. As regard *M. decipiens*, we had previously observed that infection of THP-1 cells at 37°C resulted in lower multiplication rates than at 35°C (Fig. 4A), likely due to limited growth potential at this temperature (Fig. 1A and F through G), which might also play a role in this warm-blooded *in vivo* model.

As mentioned above, the CFU counts at day 1 for *M. lacus* were higher than for the other species tested, which likely contributed to the detection of *M. lacus* in both mouse infection models at later time points. In C3HeB/FeJ mice, CFU counts were detected at 4 weeks post-infection but not at 14 weeks (Fig. 5C and D), while in C57BL/6J mice, *M. lacus* was detected in the lungs and spleens at 14 weeks post-infection but not at 4 weeks (Fig. 5G and H). These results suggest that at higher infection doses, selected members of the MTBAP, such as *M. lacus*, may multiply or persist in mouse models, although the detected CFU levels of *M. lacus* remained relatively low. Given the higher initial CFU counts for this strain found in the mouse lungs at day 1 for both mouse models, the 4- and 14-week results still point to an overall minor virulence level of *M. lacus* in mice and much lower than that of *Mtb*.

In contrast*, M. riyadhense* presented a surprisingly high *in vivo* growth profile in our infection experiments, showing a higher *in vivo* growth capacity than the other MTBAP species tested, whereby it needs to be mentioned that for the C3HeB/FeJ model, day 1 CFU counts of *M. riyadhense* were substantially higher (~500 CFUs) than those observed for the other species tested (except for *M. lacus*) (Fig. 5A). During this first experiment, *M. riyadhense*-infected mice began to lose considerable weight and had to be culled at half of the targeted time, i.e., at 7 weeks post-infection instead of 14 weeks (Fig. 5C and D). *M. riyadhense* even disseminated to the spleens of mice, like *Mtb* (Fig. 5D). However, it remained unclear how infection dynamics between *M. riyadhense* and *Mtb* might compare when similar, low initial bacterial doses were deposited in the lungs of mice. As such, the dose of CFU contained in the aerosolized solution was reduced for the second mouse infection experiment, using C57BL/6J mice. This enabled us to obtain comparable day 1 CFU counts between *M. riyadhense* and *Mtb* (~9–15 CFUs for both species) (Fig. 5E). Results from this experiment revealed that at comparable CFU counts at day 1, *M. riyadhense* showed similar *in vivo* growth characteristics as *Mtb* up to 4 weeks post-infection in the lungs and spleens of C57BL/6J mice but that at later time points, the mice seemed to control the *M. riyadhense* infection better than this was the case for infection with *Mtb*, resulting in a reduced number of CFUs of *M. riyadhense* in the lungs of mice compared to *Mtb* (Fig. 5F through H). Relative to all the other NTM species tested in this study, the *M. riyadhense* reference strain seems, however, to be clearly the most virulent one and might be the best-adapted non-BSL-3 strain for establishing a new mycobacterial virulence model in mice.

## DISCUSSION

The main aim of this study was to explore selected features concerning the genomes, evolution, and phenotypes of four NTM species that are thought to share common evolutionary features with *Mtb*, the etiological agent of human TB, and to investigate the possibility of using one or more of these NTM species as a model for the study of TB due to their easier handling. *Mtb* is a complex organism to study, notably because it is a BSL-3 pathogen, which implies the need for special equipment for its handling. To facilitate this task, scientists often use study models to elucidate various features, such as phenotypic traits and virulence mechanisms of the pathogen. For this purpose, the NTM *M. kansasii*, and *M. marinum*, which are BSL-2 organisms, are widely used and recognized as *Mtb* study models (28). Indeed, both species share many genomic features with MTBC members. *M. kansasii* has been repeatedly designated as possibly representing the environmental ancestor of *Mtb*, which after several modifications of its genome became an obligate pathogen and primarily intracellular bacterium (3, 29–31). *M. marinum* is also a closely related species to the MTBC, sharing key orthologous genes encoding virulence factors of the pathogen (2). In addition, the availability of natural infection models, such as the zebrafish or the zebrafish larvae models, has enhanced the attractivity of the *M. marinum* model of infection (32). However, certain characteristics in the evolution of *Mtb* are not shared by these two species, resulting in gaps in our understanding of the evolution of *Mtb*, rendering comparisons difficult and asking for new, closer related models (5).

To overcome these gaps, here, we hypothesized that four recently described new mycobacterial species named *M. decipiens*, *M. lacus*, *M. riyadhense*, and *M. shinjukuense* might represent novel alternative models due to their genomic and phylogenetic proximity with *Mtb* (4). Hence, we compared selected genomic features of these species with those of *Mtb* and the known comparator species *M. kansasii*, and also evaluated selected phenotypic traits and studied their infection capacity in typical models for *Mtb* virulence evaluation.

To our surprise, phenotypic characterization of the reference strains from the four species showed that *M. decipiens* differed in its optimal growth temperature compared to *Mtb* and the other three species as it showed an optimal growth temperature between 32°C and 35°C, and not 37°C, as indicated on the supplier’s data sheet or in the literature (33, 34). Results from cell and mouse infection experiments further suggested reduced bacterial fitness of *M. decipiens* at 37°C. This feature reminds of that of certain other mycobacterial species, such as *M. marinum*, which shows an optimal growth at temperatures around 30°C–32°C and no growth at 37°C (35, 36). Similarly, *Mycobacterium leprae*, the agent of leprosy, also is known to preferentially infect peripheral body parts that show lower temperature, such as the skin (37). Taken together, it seems that *M. decipiens* is sensitive to temperatures above 35°C, a feature that might be linked to its relatively low virulence in mouse infection experiments despite its close genomic relatedness to *Mtb*.

When looking at other evolutionary characteristics, such as genome size reduction, which is a characteristic feature for the evolution of pathogenic mycobacteria, *M. decipiens* shows an intermediate position. During its long-term evolution, it is hypothesized that the genome size of *Mtb* was reduced to 4.4 Mb, compared with 6.4 and 6.6 Mb for *M. kansasii* and *M. marinum*, respectively (5). Of the four investigated species, *M. riyadhense* comes closest to the genome sizes of *M. kansasii* and *M. marinum* with 6.2 Mb, followed by *M. decipiens* (5.3 Mb) and *M. lacus* (5.1 Mb), while *M. shinjukuense* exhibits a 4.5-Mb genome size that is very similar in size to that of *Mtb*. A small portion of this variable genome size reduction in the four NTM species and *Mtb* relative to *M. marinum* or *M. kansasii* is due to the absence of the pigmentation locus. Indeed *M. marinum* and *M. kansasii*, as primarily environmental organisms harbor a *crtEIB* cluster in their genomes, encoding enzymes involved in carotenoid biosynthesis (38). Genomic comparisons between *M. marinum*, *M. kansasii*, *Mtb*, and the four NTM species showed that the *crtEIB* gene cluster was not present in the genomes of the MTBAP members, suggesting that the putative common ancestor of this group of mycobacteria might not have shared the same niche as the waterborne NTM species *M. marinum* and *M. kansasii* (39).

Determining selected phenotypic characteristics of the four species also included evaluation of their putative resistance profile against established anti-TB drugs. It should be mentioned that commonly, treatment of NTM infections involves different breakpoints used for treatment of *Mtb* infections, and thus our aim here was to evaluate the putative resistance or sensitivity of the four tested species in direct comparison to *Mtb*, in a perspective to use these species as models. Our results might not be representative for the use of the tested drugs in a clinical application to treat NTM infections, which usually use different criteria and breakpoints. The tested drugs were isoniazid, rifampicin, and ethambutol, as well as bedaquiline, which is a recently developed anti-TB agent, accepted by the FDA and the EMA for use in MDR TB treatment regimens. Bedaquiline is a broad-spectrum antibiotic acting on the essential ATP synthase (40), and we found that all tested species in our study were sensitive to this potent new drug. Pyrazinamide, the fourth first-line anti-TB, was not included in this study because its activity requires acidic pH, making it difficult to test under *in vitro* conditions (41, 42). *M. decipiens* showed the largest potential resistance profile of the four species (resistance to rifampicin, ethambutol, and isoniazid), whereas *M. shinjukuense* showed potential sensitivity to all four tested drugs. While *M. decipiens* showed intermediate resistance to rifampicin, we could not detect any of the known RpoB mutations that cause resistance to rifampicin in *Mtb* (22). An alternative explication for rifampicin resistance of *M. decipiens* might be the presence of an *arr* gene encoding a rifampin ADP-ribosyl transferase in the genome of *M. decipiens*, which depicts 78.9% amino acid identity with the orthologous enzyme in *M. smegmatis*. Rifampicin ribosylation was described as a mechanism causing rifampicin resistance in *M. smegmatis* (43) and *M. abscessus* (44). Interestingly, *M. decipiens* was the only species in our study that encoded a rifampin ADP-ribosyl transferase in its genome, and *arr* orthologs are also absent from most other slow-growing mycobacteria, except for *M. marinum*, *Mycobacterium liflandii,* and *Mycobacterium ulcerans*. For the latter strains, it remains unclear if the encoded ADP-ribosyl transferases are biologically active, as *M. marinum* strains are commonly sensitive to rifampicin (45) and rifampicin is also part of the antibiotic treatment against Buruli ulcer, caused by *M. ulcerans* (46). The finding that *M. decipiens* shows resistance to rifampicin combined with the fact that it carries an *arr* gene, is thus opening new intriguing research questions and makes *M. decipiens* an interesting model to study the function of rifampin ADP-ribosyl transferases in slow-growing mycobacteria. Finally, it should be mentioned that some of the observed resistance phenomena in the NTM strains might also be caused by differences in transport or efflux of the tested anti-TB drugs, a feature to be tested in future studies involving these strains.

For studies on TB, the availability of infection models is of utmost importance. One of the most common *in vitro* models for TB research is the THP-1 cell infection model, whereas for the *in vivo* models, different mouse infection models can be used. In this work, we assessed the capacity of the MTBAP species and *M. kansasii* to multiply in cellular and in murine models. Interestingly, *M. decipiens* and *M. riyadhense* together with *Mtb* were capable of multiplication in THP-1 macrophages, but only *M. riyadhense* and *Mtb* were able to efficiently infect and replicate in mice. We want to emphasize that the results obtained for these mouse infection pilot studies represent data from single infection experiments in each particular mouse model (C3HeB/FeJ and C57BL/6J). However, even if some of the details are difficult to compare due to diverging doses observed for day 1 control mice, the general conclusion that can be drawn from these experiments is that the reference strain of *M. riyadhense* used here (DSM 45176) exhibits an impressive mouse virulence for a strain that is rated as a BSL-1 organism. This feature also enriches previous studies on evolutionary aspects of *M. riyadhense* involving clinical isolates (8). Our results are also of interest for the development of new TB pathogenicity models that might not need BSL-3 containment, although a change of the BSL-1 rating of the *M. riyadhense* reference strain to BSL-2 is strongly recommended.

To complement the virulence studies and search for plausible biological causes for the results obtained in THP-1 cells and mice, we investigated selected virulence factors thought to play an important role for *Mtb* pathogenicity. First, we focused on one well-established virulence trait of mycobacterial pathogens, which is linked to the synthesis and secretion of ESAT-6, also known as EsxA, by the ESX-1 type VII secretion (T7S) system (47, 48) that has been described to induce phagosomal rupture and necrosis in selected immune cells (14, 15, 49). Comparison of the genomic organization of the ESX-1 loci in the four species showed that *M. decipiens* shares a higher similarity in the ESX-1 proteins with *Mtb* than the other species. Indeed, most *M. decipiens* ESX-1 proteins show ~90% or greater amino acid identity with the orthologous proteins in *Mtb*, which is about 10% higher than the commonly observed ~80% seen for the other species, including *M. kansasii*. As described in the Results section, secretion of ESAT-6 (EsxA) and its partner protein CFP-10 (EsxB) was evaluated by immunoblotting under two conditions, first in cultures that were grown to OD_600nm_ of 0.6–0.8 and second in cultures grown to higher ODs, greater than 1. Interestingly, ESAT-6 secretion was found in *M. decipiens* under both conditions, whereas for the other NTM species, we only detected a small amount of ESAT-6 in the supernatant of *M. riyadhense* under high-density culture conditions. In contrast, CFP-10 was detected in the supernatants of all NTM under high-density culture conditions. It is tempting to speculate that the difference between CFP-10 and ESAT-6 secretion in some NTM might in part be linked to the absence of an *espACD* orthologous locus in these species, which in *Mtb* represents an essential constituent for co-secretion of ESAT-6, CFP-10, EspA, and EspC (16–18). Interestingly, the finding that CFP-10 is secreted without its usual protein partner ESAT-6 is reminiscent of the situation observed in the MTBVAC vaccine strain, which represents a virulence-attenuated *Mtb* strain that is deleted for the two-component regulator PhoP (50). As from previous studies it is known that PhoP indirectly regulates the *espACD* cluster (51), the observed differences in the secretion profiles of CFP-10 and ESAT-6 for several NTM confirm observations with the MTBVAC strain and suggest that ESAT-6 and CFP-10 may not always be secreted as a 1:1 complex, which represented a long-lasting paradigm of ESX-1 secretion (52, 53). Our results with *M. riyadhense* also suggest that *in vitro* generated secretion profiles may change with culture density. For the moment, it is unknown if the *in vitro* secretion profile of such strains also applies to the *in vivo* situation. Immunologic studies that have the potential to provide responses to these questions (54) will be needed to get further insights into this matter.

In conclusion, our study has elucidated genomic and phenotypic particularities of members of the MTBAP that provide a foundation on which to expand the use of these strains in TB research. As shown, each of the NTM strains tested has unique characteristics that should prove useful for exploring specific, but as yet unanswered, biological questions in the evolution, virulence, and drug susceptibility of tubercle bacilli. These NTM strains will therefore contribute to expanding our available toolset and to advancing the TB research field.

## MATERIALS AND METHODS

### Purchase and culture of strains

Reference strains belonging to the species *M. decipiens* (ATCC TSD-117), *M. lacus* (DSM 44577), *M. riyadhense* (DSM 45176), and *M. shinjukuense* (DSM 45663) were purchased from the American Type Culture collection (ATCC) and German Collection of Microorganisms and Cell Cultures (DSM), respectively.

Mycobacterial strains were cultured in liquid Middlebrook 7H9 media (Difco) supplemented with 10% albumin-dextrose-catalase (Difco) and 0.05% Tween 80, and on Middlebrook 7H11 agar media (Difco) supplemented with 10% oleic acid-albumin-dextrose-catalase (Difco) at 37°C, unless otherwise specified.

### DNA extraction and genome sequencing

Pellets of cultures in exponential phase were recovered, and 5 mL of solution I (saccharose 25%, EDTA 50 mM pH 8, Tris-HCl 50 mM pH 8, Thiourea 50 mM, H20) with 250 µL of lysozyme 10 mg/mL was added. Solutions were mixed and incubated 4 hours at 37°C. Furthermore, 5 mL of solution II (SDS 1%, Tris-HCl 100 mM pH 8, proteinase K 0.4 mg/mL, H20) was added and mixed. Solutions were transferred into 10 mL Nalgene bottles (Fisher Scientific) with 2.5 mL of zirconium beads, shaken three times at 30 Hz for 3 min, and incubated overnight at 65°C. Solutions were transferred without the beads, and NaCl solution was added at a final concentration of 0.625 M and mixed by inversion. The aqueous phases were transferred into new tubes after centrifugation at 5,000 g for 10 min. Then, 5 mL of phenol/chloroform/isoamyl alcohol was added to the aqueous phases, mixed, and centrifuged at 5,000 g for 10 min. The resulting new aqueous phases were transferred into new tubes, and two volumes of ice-cold ethanol 99% were added and mixed by inversion before being incubated at −20°C for at least 2 hours. The tubes were then centrifuged at 10,000 g for 30 min at 4°C, and supernatants were discarded. Pellets were washed once with 5 mL of cold ethanol 70% by adding the ethanol onto the pellets followed by centrifugation at 10,000 g for 10 min at 4°C. Supernatants were discarded again, and pellets were dried and resuspended in 1× TE buffer. The quality and concentration of each DNA sample were then analyzed using the Qbit dsDNA Quantification Assay (Invitrogen).

### Genome sequencing using Pacific Biosciences technologies

High-molecular weight input DNA was purified with magnetic beads to a level of 0.45× before being fragmented a Covaris G-Tube targeting a fragment size of 10 kb. A SMRTbell Express Template Prep Kit 2.0 was used. For sequencing, a Sequel Binding Kit 3.0 was used together with a Sequel DNA Internal Control 3.0 and a Sequel Sequencing Plate 3.0. Sample loading concentration for the Sequel I sequencer was 10 pM. A sequencing mode for continuous long reads was used for 10 hours. A software version of SMRT Link: 9.0.0.92188; Chemistry Bundle: 9.0.0.92017; Params: 9.0.0 was used.

### Genome sequencing using Nanopore technologies

High-molecular weight input DNA was purified with magnetic beads to a level of 0.45× and then fragmented with a Megaruptor 3, using a low volume protocol (65 µL), targeting fragment sizes of 10–15 kb. A ligation sequencing gDNA native barcoding v14 sqk nbd114-24 NBE 9169 preparation kit was used, with elimination of fragments smaller than 3 kb. A sequence-ready library was generated and loaded on a FLO-MIN114 (R10.4.1) flow cell. A 72-hour sequencing protocol was initiated using the MinKNOW (version 22.07.9). The actual sequencing duration was then 23 hours, using a translocation speed of 260, a minimal read length of 200 bp, and the high-accuracy base-calling mode.

### Genomic comparisons

Genomic comparisons of ESX loci of selected NTM with *Mtb* and *M. kansasii* were performed using the Artemis Comparison Tool ACT (55) and the “MicroScope” (9) database from the Genoscope.

### Antibiotic resistance assay

Mycobacterial cultures in exponential phase were washed several times with phosphate-buffered saline (PBS), and clumps were broken by passing the culture through 10 µm filters (Pluriselect). Bacterial suspensions were used at a final OD_600nm_ of 0.005 in 96-well plates. Antibiotics were added to bacterial solutions and diluted to have a range of concentrations as follow: bedaquiline (3.2–1.6–0.8–0.4–0.2–0.1–0.05–0.025 –0.0125–0.00625–0.00313–0.00156–0.00078–0.00039 µg/mL); isoniazid (32–16–8–4–2–1–0.5–0.25–0.125–0.0625–0.0313–0.0156–0.0078 µg/mL); ethambutol (60–30–15–7.5–3.75–1.88–0.94–0.47–0.23–0.12 µg/mL); and rifampicin (4–2–1–0.5–0.25–0.125–0.0625–0.0313–0.0156–0.0078–0.0039–0.002 µg/mL). Plates were incubated 9 days at 37°C for *M. lacus*, 10 days for *M. riyadhense*, 12 days for *M. shinjukuense*, 6 days for *M. kansasii* and *Mtb,* and 8 days at 35°C for *M. decipiens*. After the incubation period, 30 µL of 0.01% resazurin solution (resazurin sodium salt, Sigma) was added and incubated at the same temperature as previously used. Plates were read with an excitation wavelength of 570 nm and emission of 590 nm.

### THP-1 macrophages infection

Human monocyte cell line THP-1 cells (ATCC-TIB-202) were cultured at 37°C and 5% CO_2_ in RPMI-1640 GlutaMAX (Gibco) medium supplemented with 10% of inactivated fetal bovine serum (Dutsher). Cells were seeded at a density of 1 × 10^5^ cells/100 µL in 96-well plates (TPP) and differentiated into macrophages using phorbol-myristate-acetate (Sigma) at 10 ng/mL for 48 hours. Bacterial solutions were prepared and added on macrophages at an MOI of 0.05 (1 bacterium for 20 THP1 cells) for 3 hours, and extracellular bacteria were eliminated by amikacin treatment at 0.1 mg/mL for 1 hour. Plates were incubated for 6 days at 35°C and 37°C with 5% of CO_2_. Capacity of infection was determined by CFU counting after 0, 3, 5, and 7 days of infection using 0.1% Triton-X100 to lyse THP-1 cells.

### Western blotting

Bacterial cultures were grown in initial experiments until an OD_600nm_ of 0.6–0.8 and in additional experiments to ODs higher than 1. To avoid reaction with albumin, cultures were washed and incubated for 48 hours in 7H9 media supplemented with 0.2% dextrose and 0.05% Tween 80. Supernatants were recovered and treated with protease inhibitor cocktails (cOmplete EDTA-free protease inhibitor, Sigma) before being filtered through a 0.22-µm filter. Cell pellets were washed with PBS and treated with the protease inhibitor cocktails. Cell membranes were lysed using TissueLyser II (Qiagen) for 8 min at 30 Hz.

An amount of 50 µg of proteins from cell lysate and supernatant samples was separated by SDS-PAGE on NuPage 10% Bis-Tris gels (Invitrogen) and transferred on a nitrocellulose membrane with the iBlot dry blotting system (Invitrogen). Immunodetection was performed using anti-ESAT-6 antibodies [produced in-house (56)], anti-CFP-10 antibodies (a kind gift from I. Rosenkrands, Statens Serum Institut, Copenhagen, Denmark), anti-SigA antibodies (a kind gift from I. Rosenkrands, Statens Serum Institut, Copenhagen, Denmark), and commercial polyclonal anti-Ag85B antibodies (Abcam, ab43019). All antibodies were polyclonal and used at 1/5,000 in TBS 0.1% Tween 20 and 3% BSA. Revelation was made using the SuperSignal West Femto Maximum Sensitivity Substrate (Thermo Fisher).

### Mouse infection

Female C57BL/6J (Charles River) mice or male C3HeB/FeJ mice from an Institute-intern breeding facility were purchased and transferred in dedicated isolators inside a BSL-3 facility. After an adaption phase of several days, groups of mice were infected by aerosol with 5 mL of 2.5 × 10^6^ bacteria solutions using a custom-made aerosolization system. The final doses administered to the groups of mice were assessed by CFU quantification in lung homogenates obtained from control mice at day 1 post-infection, in addition to plating the dilutions of the solutions used, just after the aerosol challenge. The *in vivo* growth capacity of each strain was determined by plating homogenized mouse lungs and spleens at 4 and 14 weeks post-infection and determining CFU counts. Mice were monitored on a daily basis and culled in the case of 20% weight loss.

## Data Availability

Long-read sequence data have been deposited in the European Nucleotide Archive under accession PRJEB70771.

## SUPPLEMENTAL MATERIAL

Fig. S1 spectrum.04126-23-s0001.pdf Comparison of the ESX systems in NTM.

Fig. S2 spectrum.04126-23-s0002.pdf Analysis of ESX-2 system in selected species.

Fig. S3 spectrum.04126-23-s0003.pdf Results from the reazurin assay.

ASM does not own the copyrights to Supplemental Material that may be linked to, or accessed through, an article. The authors have granted ASM a non-exclusive, world-wide license to publish the Supplemental Material files. Please contact the corresponding author directly for reuse.
