# Supplementary material for: Genomic and phenotypic characterization of Mycobacterium tuberculosis’ closest-related non-tuberculous mycobacteria
Source: Microbiol Spectr. 2024 May 3;12(6):e04126-23. doi: 10.1128/spectrum.04126-23 (PMC11237670; doi:10.1128/spectrum.04126-23)
Supplement: Fig. S1 — Comparison of the ESX systems in NTM. [file spectrum.04126-23-s0001.pdf]

Supplemental Fig. S1: Comparison of the ESX systems of *M. lacus, M. riyadhense*, *M. shinjukuense* with *M. kansasii* and *Mtb* H37Rv.
Comparisons were performed for ESX-1 (A), ESX-2 (B), ESX-3 (C), ESX-4 (D), and ESX-5 (E) using the Artemis Comparison Tool and the "MicroScope" database from the Genoscope. (https://mage.genoscope.cns.fr/microscope/home/index.php)

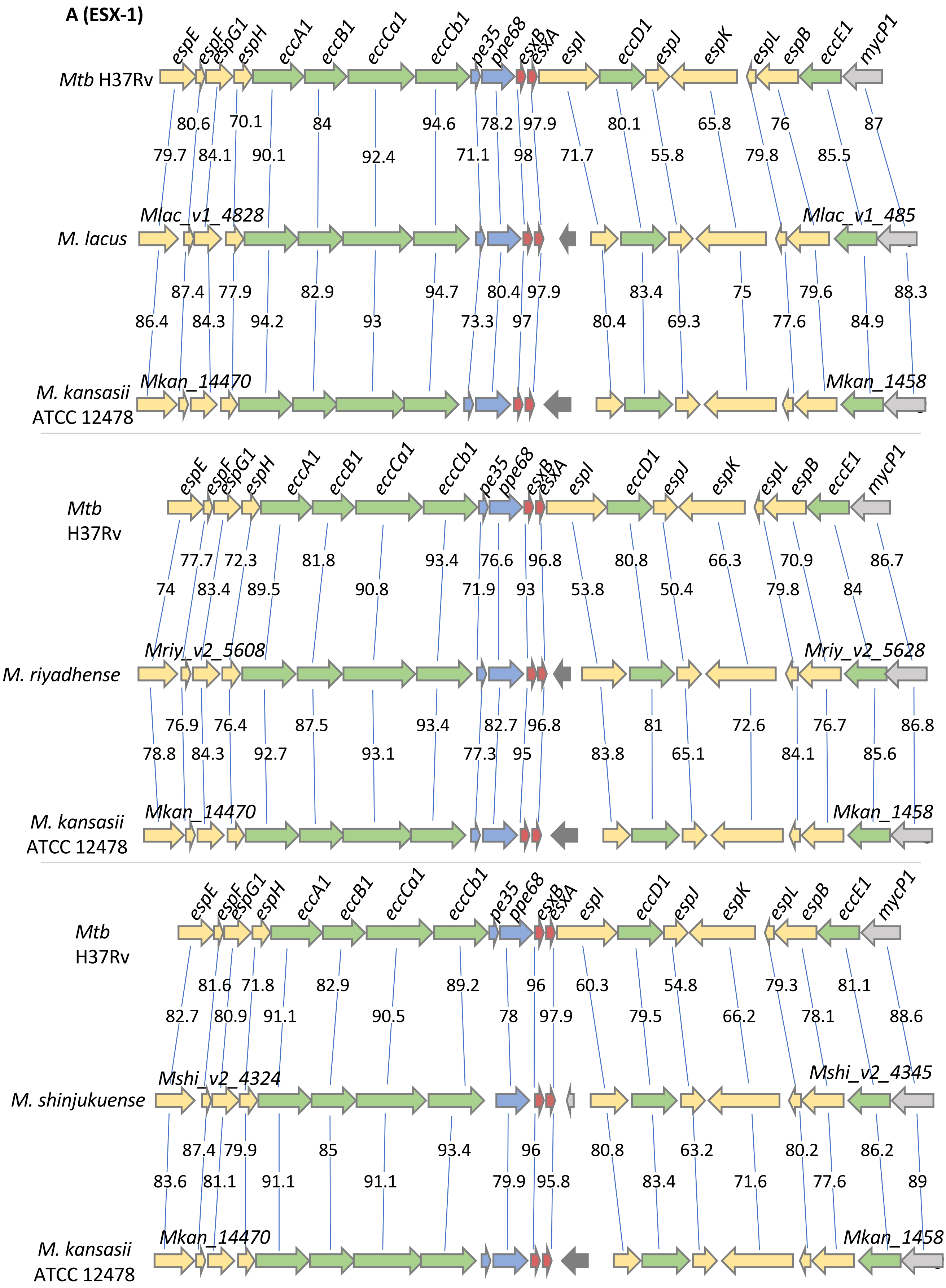

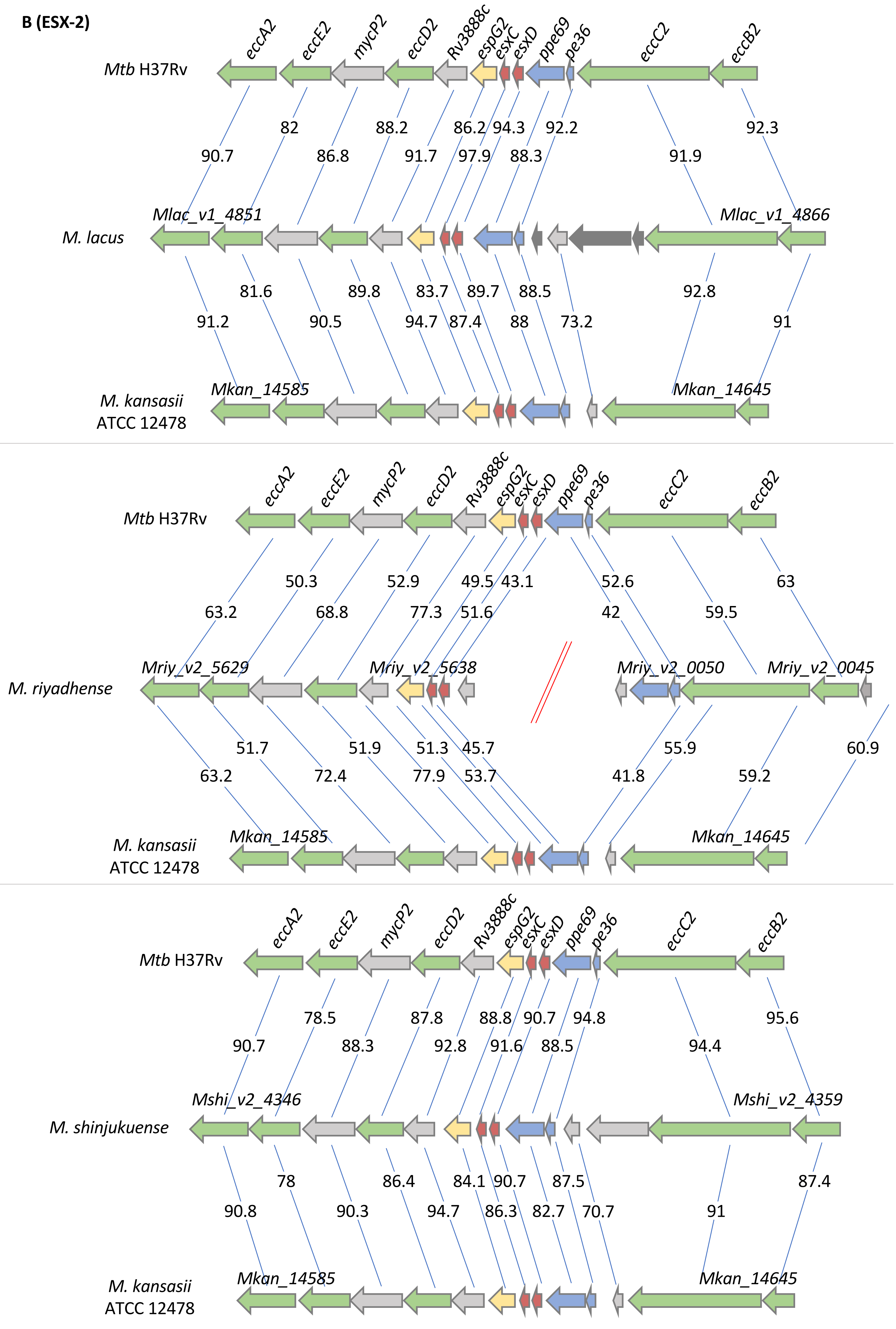
B (ESX-2)
Mtb H37Rv
eccA2
eccE2
mycP2
eccD2
Rv3888c
espG2
esxC
esxD
ppe69
pe36
eccC2
eccB2
90.7
82
86.8
88.2
91.7
86.2
94.3
97.9
88.3
92.2
91.9
92.3
M. lacus
Mlac_v1_4851
Mlac_v1_4866
91.2
81.6
90.5
89.8
94.7
83.7
87.4
89.7
88
88.5
73.2
92.8
91
M. kansasii ATCC 12478
Mkan_14585
Mkan_14645
Mtb H37Rv
eccA2
eccE2
mycP2
eccD2
Rv3888c
espG2
esxC
esxD
ppe69
pe36
eccC2
eccB2
63.2
50.3
68.8
52.9
77.3
49.5
43.1
51.6
52.6
42
59.5
63
M. riyadhense
Mriy_v2_5629
Mriy_v2_5638
Mriy_v2_0050
Mriy_v2_0045
63.2
51.7
72.4
51.9
77.9
51.3
45.7
53.7
41.8
55.9
59.2
60.9
M. kansasii ATCC 12478
Mkan_14585
Mkan_14645
Mtb H37Rv
eccA2
eccE2
mycP2
eccD2
Rv3888c
espG2
esxC
esxD
ppe69
pe36
eccC2
eccB2
90.7
78.5
88.3
87.8
92.8
88.8
90.7
91.6
88.5
94.8
94.4
95.6
M. shinjukuense
Mshi_v2_4346
Mshi_v2_4359
90.8
78
90.3
86.4
94.7
84.1
90.7
86.3
82.7
87.5
70.7
91
87.4
M. kansasii ATCC 12478
Mkan_14585
Mkan_14645

C (ESX-3)

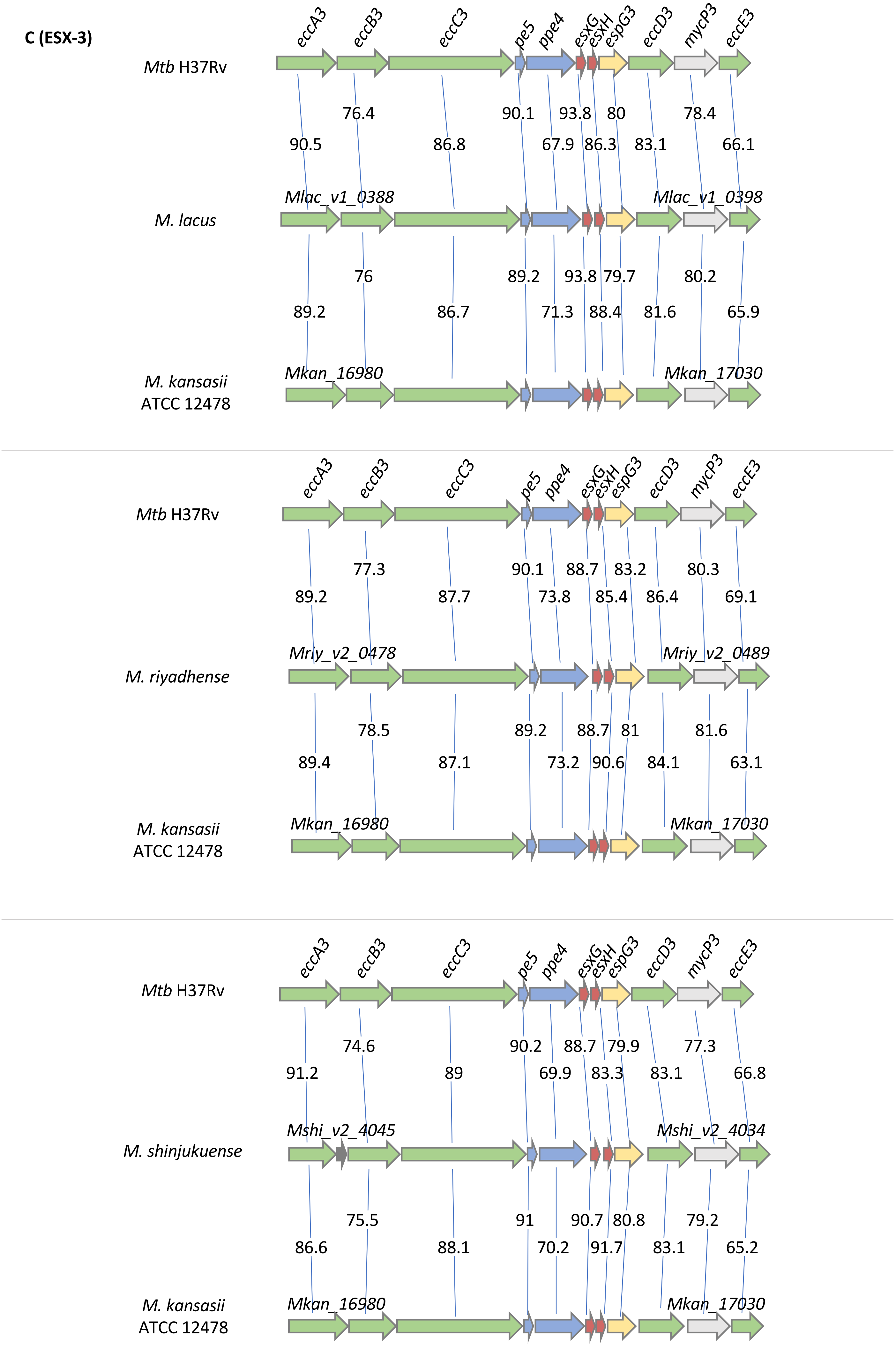
eccA3
eccB3
eccC3
pe5
ppe4
esxG
esxH
espG3
eccD3
mycP3
eccE3
Mtb H37Rv
76.4
90.1
93.8
80
78.4
90.5
86.8
67.9
86.3
83.1
66.1
Mlac_v1_0388
Mlac_v1_0398
M. lacus
76
89.2
93.8
79.7
80.2
89.2
86.7
71.3
88.4
81.6
65.9
Mkan_16980
Mkan_17030
M. kansasii ATCC 12478
eccA3
eccB3
eccC3
pe5
ppe4
esxG
esxH
espG3
eccD3
mycP3
eccE3
Mtb H37Rv
77.3
90.1
88.7
83.2
80.3
89.2
87.7
73.8
85.4
86.4
69.1
Mriy_v2_0478
Mriy_v2_0489
M. riyadhense
78.5
89.2
88.7
81
81.6
89.4
87.1
73.2
90.6
84.1
63.1
Mkan_16980
Mkan_17030
M. kansasii ATCC 12478
eccA3
eccB3
eccC3
pe5
ppe4
esxG
esxH
espG3
eccD3
mycP3
eccE3
Mtb H37Rv
74.6
90.2
88.7
79.9
77.3
91.2
89
69.9
83.3
83.1
66.8
Mshi_v2_4045
Mshi_v2_4034
M. shinjukuense
75.5
91
90.7
80.8
79.2
86.6
88.1
70.2
91.7
83.1
65.2
Mkan_16980
Mkan_17030
M. kansasii ATCC 12478

D (ESX-4)

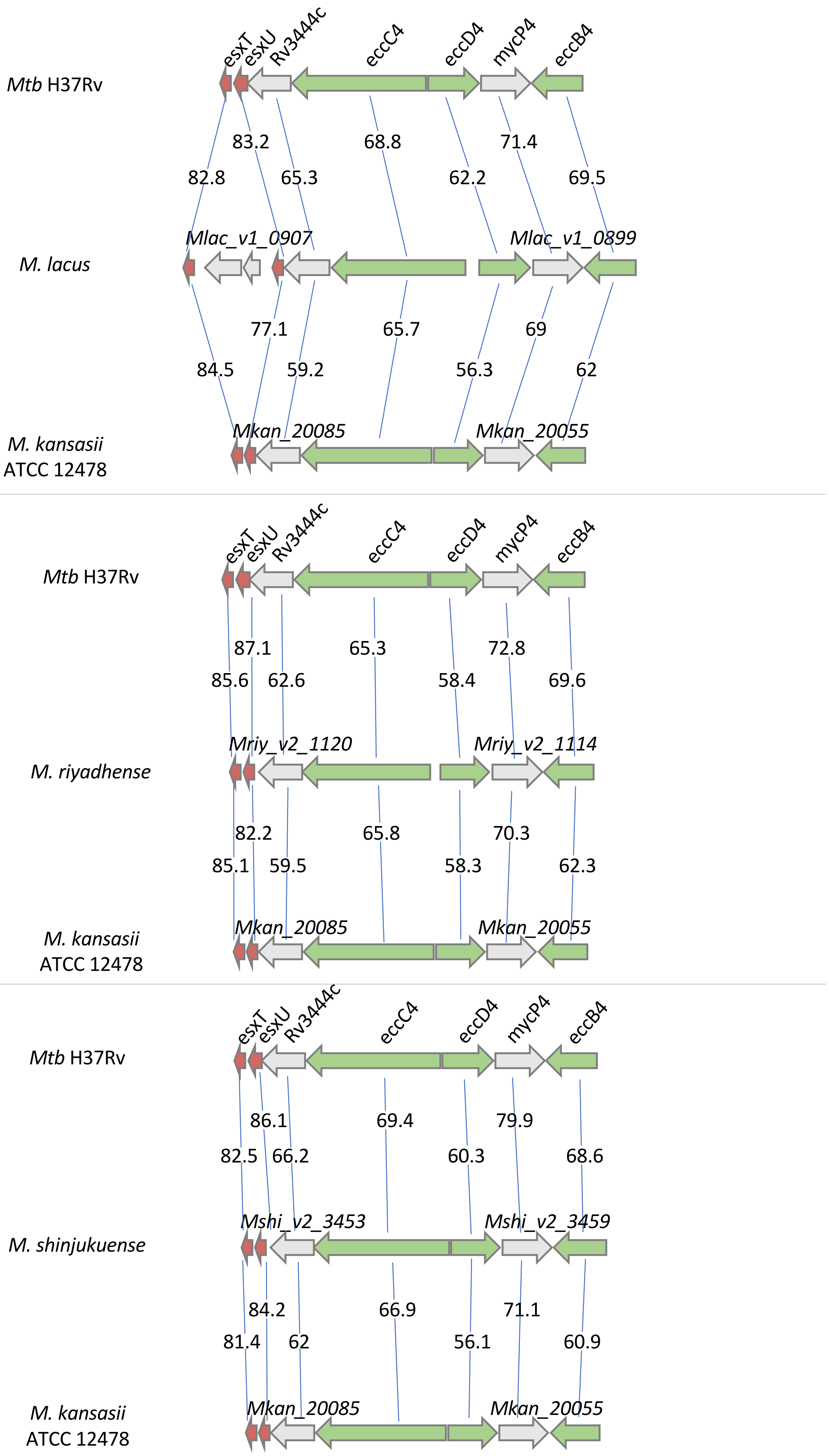

Mtb H37Rv
esxT
esxU
Rv3444c
eccC4
eccD4
mycP4
eccB4
83.2
68.8
71.4
82.8
65.3
62.2
69.5
M. lacus
Mlac_v1_0907
Mlac_v1_0899
77.1
65.7
69
84.5
59.2
56.3
62
M. kansasii
ATCC 12478
Mkan_20085
Mkan_20055
Mtb H37Rv
esxT
esxU
Rv3444c
eccC4
eccD4
mycP4
eccB4
87.1
65.3
72.8
85.6
62.6
58.4
69.6
M. riyadhense
Mriy_v2_1120
Mriy_v2_1114
82.2
65.8
70.3
85.1
59.5
58.3
62.3
M. kansasii
ATCC 12478
Mkan_20085
Mkan_20055
Mtb H37Rv
esxT
esxU
Rv3444c
eccC4
eccD4
mycP4
eccB4
86.1
69.4
79.9
82.5
66.2
60.3
68.6
M. shinjukuense
Mshi_v2_3453
Mshi_v2_3459
84.2
66.9
71.1
81.4
62
56.1
60.9
M. kansasii
ATCC 12478
Mkan_20085
Mkan_20055

E (ESX-5

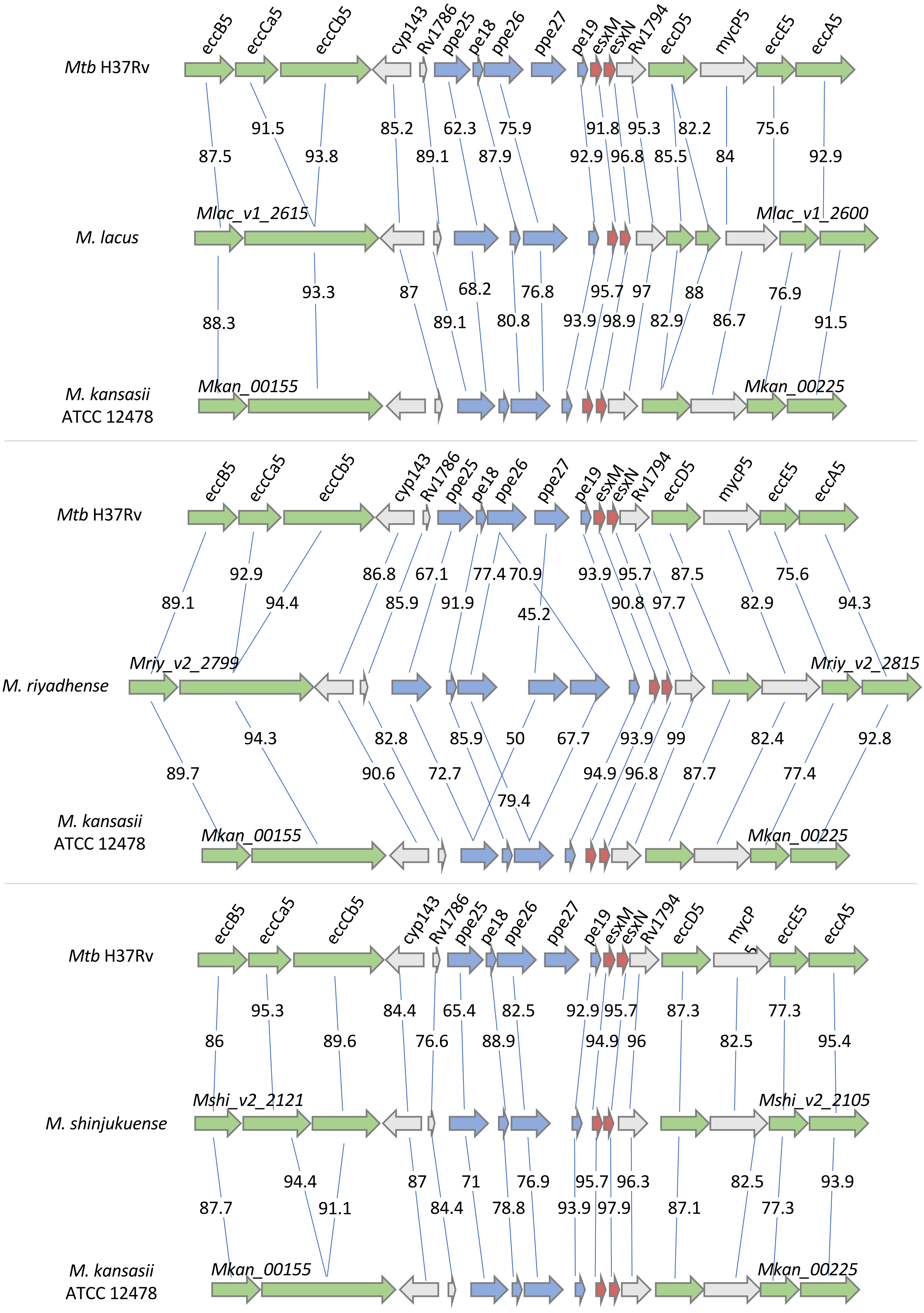
eccB5
eccCa5
eccCb5
cyp143
Rv1786
ppe25
pe18
ppe26
ppe27
pe19
esxM
esxN
Rv1794
eccD5
mycP5
eccE5
eccA5
Mtb H37Rv
91.5
85.2
62.3
75.9
91.8
95.3
82.2
75.6
87.5
93.8
89.1
87.9
92.9
96.8
85.5
84
92.9
Mlac_v1_2615
Mlac_v1_2600
M. lacus
93.3
87
68.2
76.8
95.7
97
88
76.9
88.3
89.1
80.8
93.9
98.9
82.9
86.7
91.5
M. kansasii
ATCC 12478
Mkan_00155
Mkan_00225
eccB5
eccCa5
eccCb5
cyp143
Rv1786
ppe25
pe18
ppe26
ppe27
pe19
esxM
esxN
Rv1794
eccD5
mycP5
eccE5
eccA5
Mtb H37Rv
92.9
86.8
67.1
77.4
70.9
93.9
95.7
87.5
75.6
89.1
94.4
85.9
91.9
45.2
90.8
97.7
82.9
94.3
Mriy_v2_2799
Mriy_v2_2815
M. riyadhense
94.3
82.8
85.9
50
67.7
93.9
99
82.4
92.8
89.7
90.6
72.7
79.4
94.9
96.8
87.7
77.4
M. kansasii
ATCC 12478
Mkan_00155
Mkan_00225
eccB5
eccCa5
eccCb5
cyp143
Rv1786
ppe25
pe18
ppe26
ppe27
pe19
esxM
esxN
Rv1794
eccD5
mycP
5
eccE5
eccA5
Mtb H37Rv
95.3
84.4
65.4
82.5
92.9
95.7
87.3
77.3
86
89.6
76.6
88.9
94.9
96
82.5
95.4
Mshi_v2_2121
Mshi_v2_2105
M. shinjukuense
94.4
87
71
76.9
95.7
96.3
82.5
93.9
87.7
91.1
84.4
78.8
93.9
97.9
87.1
77.3
M. kansasii
ATCC 12478
Mkan_00155
Mkan_00225
