# Supplementary material for: Genomic and phenotypic characterization of Mycobacterium tuberculosis’ closest-related non-tuberculous mycobacteria
Source: Microbiol Spectr. 2024 May 3;12(6):e04126-23. doi: 10.1128/spectrum.04126-23 (PMC11237670; doi:10.1128/spectrum.04126-23)

**Supplemental Fig. S2:** Analysis of ESX-2 system sections of *M. decipiens* and *M. riyadhense* by PCR. The two sections of the ESX-2 locus in *M. decipiens* (A) and *M. riyadhense* (B) are represented with the amplified PCR fragment annotated (C). The amplified fragments 1, 2, and 3 (C) were obtained with *M. decipiens* template DNA (two different samples), while the amplified fragments 4, 5, 6, and 7 (C) were obtained by using template DNA from *M. riyadhense*.

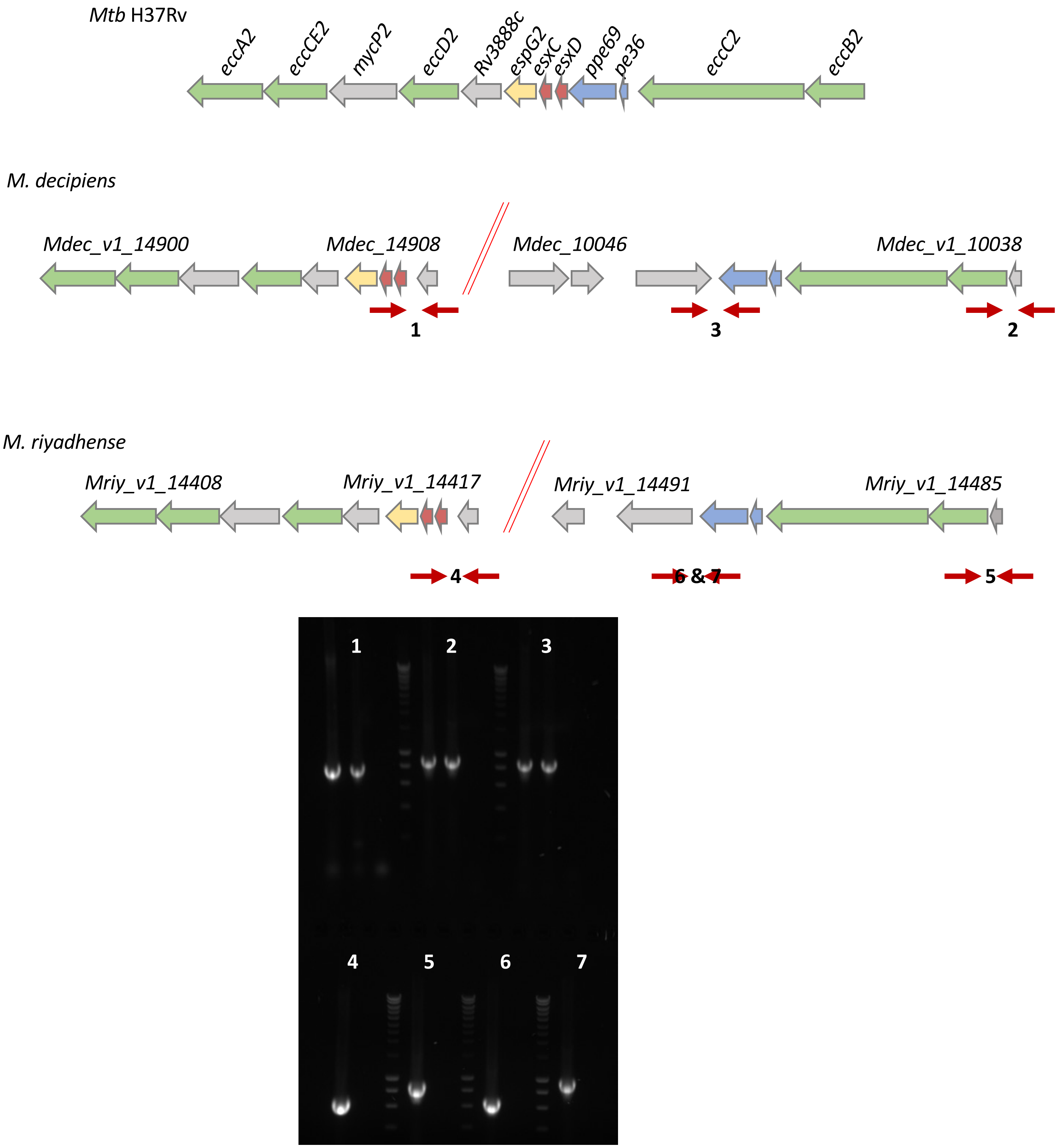

Supplement: Fig. S2 — Analysis of ESX-2 system in selected species. [file spectrum.04126-23-s0002.pdf]
