# Supplementary material for: Genomic and phenotypic characterization of Mycobacterium tuberculosis’ closest-related non-tuberculous mycobacteria
Source: Microbiol Spectr. 2024 May 3;12(6):e04126-23. doi: 10.1128/spectrum.04126-23 (PMC11237670; doi:10.1128/spectrum.04126-23)

Supplemental Fig. S3: Results of resazurin assays to determine the MIC and the resistance to anti-TB drugs of the four tested NTM species, and *M. kansasii* and *Mtb* H37Rv as controls.

Plate layout:

µg/mL											Range (% OD)	
RIF	4	2	1	0.5	0.25	0.125	0.0625	0.0313	0.0156	0.0078	100	100
	4	2	1	0.5	0.25	0.125	0.0625	0.0313	0.0156	0.0078	90	90
INH	32	16	8	4	2	1	0.5	0.25	0.125	0.0625	75	75
	32	16	8	4	2	1	0.5	0.25	0.125	0.0625	50	50
ETH	60	30	15	7.5	3.75	1.875	0.9375	0.4688	0.2344	0.1172	10	10
	60	30	15	7.5	3.75	1.875	0.9375	0.4688	0.2344	0.1172	5	5
BDQ	0.2	0.1	0.05	0.025	0.0125	0.00625	0.00313	0.00156	0.00078	0.00039	2	2
	0.2	0.1	0.05	0.025	0.0125	0.00625	0.00313	0.00156	0.00078	0.00039	0	0

M. decipiens

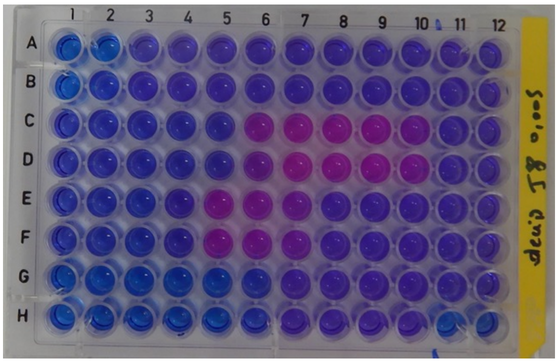

M. lacus

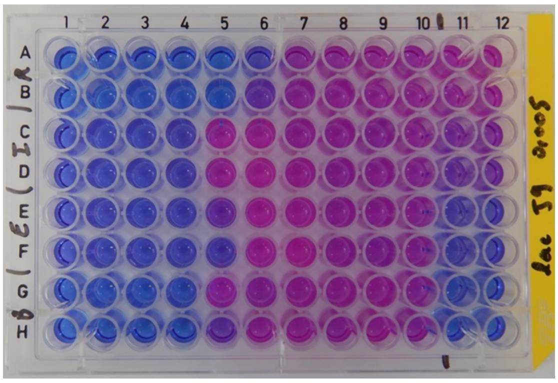

M. riyadhense

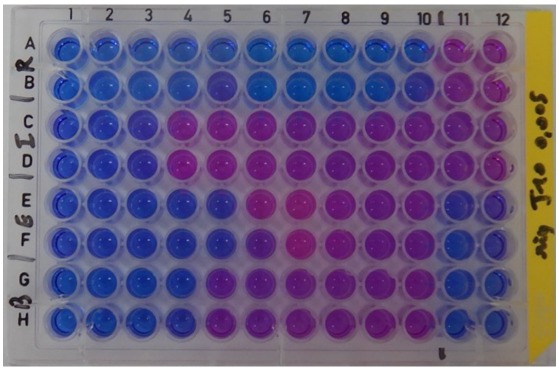

M. shinjukuense

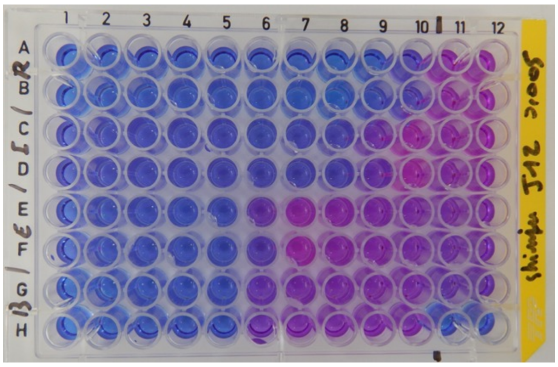

M. kansasii

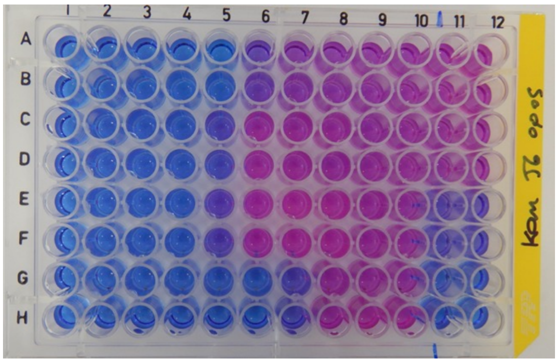

Mtb H37Rv

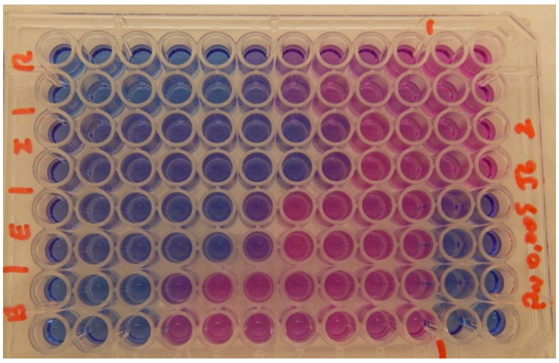

Supplement: Fig. S3 — Results from the reazurin assay. [file spectrum.04126-23-s0003.pdf]
